# Cross-sectional imaging of complicated urinary infections affecting the lower tract and male genital organs

**DOI:** 10.1007/s13244-016-0503-8

**Published:** 2016-06-07

**Authors:** Massimo Tonolini, Sonia Ippolito

**Affiliations:** Department of Radiology, “Luigi Sacco” University Hospital, Via G.B. Grassi 74, 20157 Milan, Italy

**Keywords:** Lower urinary tract, Urinary tract infection, Urinary sepsis, Computed tomography (CT), Magnetic resonance imaging (MRI)

## Abstract

Complicated urinary tract infections (C-UTIs) are those associated with structural or functional genitourinary abnormalities or with conditions that impair the host defence mechanisms, leading to an increased risk of acquiring infection or failing therapy. C-UTIs occur in patients with risk factors such as neurogenic dysfunction, bladder outlet obstruction, obstructive uropathy, bladder catheterisation, urologic instrumentation or indwelling stent, urinary tract post-surgical modifications, chemotherapy- or radiation-induced damage, renal impairment, diabetes and immunodeficiency.

Multidetector CT and MRI allow comprehensive investigation of C-UTIs and systemic infection from an unknown source. Based upon personal experience at a tertiary care hospital focused on the treatment of infectious illnesses, this pictorial essay reviews with examples the clinical features and cross-sectional imaging findings of C-UTIs affecting the lower urinary tract and male genital organs. The disorders presented include acute infectious cystitis, bladder mural abscesses, infections of the prostate and seminal vesicles, acute urethritis and related perineal abscesses, funiculitis, epididymo-orchitis and scrotal abscesses. Emphasis is placed on the possible differential diagnoses of lower C-UTIs.

The aim is to provide radiologists greater familiarity with these potentially severe disorders which frequently require intensive in-hospital antibiotic therapy, percutaneous drainage or surgery.

*Teaching Points*

• *Complicated urinary tract infections occur in patients with structural or functional risk factors*.

• *CT and MRI comprehensively investigate complicated urinary infections and sepsis from unknown sources*.

• *Infections of the urinary bladder, prostate, seminal vesicles, urethra and scrotum are presented*.

• *Emphasis is placed on differential diagnoses of complicated lower urogenital infections*.

• *Unsuspected urinary infections may be detected on CT performed for other clinical reasons*.

## Introduction

### Background

Worldwide, urinary tract infections (UTIs) account for hundreds of thousands of outpatient visits and emergency and hospital admissions each year. The vast majority of UTIs result from ascending infection from the urethra; alternatively, micro-organisms sometimes reach the urinary tract through haematogenous or lymphatic spread. UTIs represent the commonest (almost 40 %) type of hospital-acquired infections, with bladder catheterisation and urologic instrumentation as crucial risk factors [1].

According to the guidelines issued by the European Association of Urology (EAU), complicated UTIs (C-UTIs) are those associated with structural or functional abnormalities of the genitourinary tract, or with the presence of an underlying disease that interferes with host defence mechanisms, resulting in increased risk of acquiring infection or failing therapy. Enterobacteriaceae such as *E. coli* are the predominant causative organisms (approximately 65–70 % of infections), but the spectrum of pathogens is much wider than that in uncomplicated UTI, and bacteria are more likely to be resistant to antibiotics [1].

### Purpose

Over the last decade, technical advances in computed tomography (CT) and magnetic resonance imaging (MRI) have increasingly allowed comprehensive high-resolution assessment of genitourinary structures and disorders. As a result, cross-sectional imaging is increasingly used to investigate C-UTIs and systemic infection from an unknown cause [2, 3]. However, the radiological literature describing the CT and MRI features of C-UTIs affecting the lower urogenital structures is limited [4–6].

Based upon our personal experience at a tertiary care hospital focused on treatment of infectious illnesses, this pictorial essay reviews with examples the imaging findings of C-UTIs involving the lower urinary and male genital tract, aiming to provide radiologists greater familiarity with these disorders and with their differential diagnoses.

## Clinical overview of urinary tract infections

The commonest conditions predisposing patients to either acquiring infection or experiencing a more severe outcome are listed in Table 1, categorised with the mnemonic RENUC [1].

**Table 1:** Risk factors for acquiring urinary tract infection, developing complications, and/or failing treatment (mnemonic RENUC) [Adapted from Ref. 1]

Type	Risk factors	Risk of more severe outcome
Recurrent	Sexual behaviour Contraceptive devices Post-menopausal hormonal deficiency Controlled diabetes mellitus	No
Extra-urogenital	Pregnancy Male gender Badly controlled diabetes Immunosuppression including HIV, uremia, transplant recipients, treatment with corticosteroids, chemotherapy or immunosuppressants Connective tissue disease	Yes
Nephropathy	Impaired renal function Polycystic kidney	Yes
Urological	Obstructive uropathy, e.g. congenital, lithiasis, stricture, tumour Short-term catheterisation Neurogenic bladder Urological surgery or instrumentation	Yes
Permanent Catheter or non-resolvable urological risk factors	Long-term catheter Non-resolvable obstruction Badly controlled neurogenic bladder	Yes

C-UTIs may or may not cause symptoms. Manifestations of active infection often overlap with pre-existing chronic complaints secondary to the underlying lower urinary tract dysfunction. The severity of UTI is graded clinically as follows: a) asymptomatic; b) causing local symptoms such as dysuria, urinary frequency, urgency, supra- or retropubic pain or bladder tenderness; c) causing general symptoms including fever, flank pain, nausea and vomiting; d) systemic inflammatory response syndrome (SIRS); and e) circulatory and organ failure. The SIRS includes fever or hypothermia, leukocytosis or leukopenia, tachycardia and tachypnoea, and represents the first step to multi-organ failure. Urosepsis is defined by the presence of UTI plus SIRS, and warrants immediate bacteriological and imaging investigation to allow for timely treatment. Although associated with a better prognosis than other forms of sepsis, urosepsis remains a critical situation, with a 20-40 % fatality rate, particularly in the elderly and immunocompromised. Mortality is considerably increased in severe sepsis (with symptoms of organ dysfunction) and septic shock (with persistent hypotension) [1, 7].

## Indications and techniques for imaging urinary tract infections

Uncomplicated UTIs, particularly those commonly occurring in otherwise healthy young women, generally do not require further investigation apart from laboratory and microbiological studies. Conversely, nowadays, imaging is generally warranted in patients with recurrent infections or C-UTI and in those who do not respond to antibiotic therapy. The key aims of imaging in urinary infections and urosepsis are a) confirmation of urological cause, b) detection of obstruction and abscesses requiring interventional or surgical treatment, and c) detection of urolithiasis and retained foreign bodies such as catheters [1, 2, 6].

### Role of ultrasound

Non-invasive, inexpensive and repeatable, ultrasound is the mainstay first-line investigation, providing a rapid overview of the entire urinary tract, with successful detection of urinary obstruction and most abnormal collections. The initial diagnostic assessment of C-UTIs affecting the lower urinary and male genital tract should rely on transabdominal urinary tract sonography. Colour Doppler ultrasound (CD-US) with high-resolution linear probes is warranted when symptoms and physical signs suggest involvement of the scrotum and perineum. Furthermore, if allowed by local pain and tenderness, transrectal ultrasound (TRUS) may offer rapid assessment for the presence of abnormal masses or collections in the prostate and seminal vesicles. Finally, sonography often allows for an easier, non-invasive post-treatment follow-up of abnormalities detected at cross-sectional imaging [4, 8, 9].

### Role of CT

Multidetector CT reliably provides a panoramic visualisation of the entire abdomen and pelvis, and represents the mainstay modality for the detection of intra-abdominal and urological infections in the majority of patients and situations. CT is superior to ultrasound in its ability to consistently identify calcific stones along the entire urinary system and to identify gaseous collections, and may detect inflammatory changes in the perivisceral fat and abnormal contrast enhancement in inflamed tissues, urothelium and abscess walls [4–6].

The main indications for obtaining CT scans are suspected or confirmed urosepsis and sepsis from an unknown source: in this setting, CT offers a proven benefit in detecting the infectious focus and the possible underlying structural abnormalities of the urinary tract. In our experience, CT is generally useful, and may thus be recommended in patients with high or persistent suspicion of lower urogenital tract infection despite inconclusive ultrasound findings, and in those patients with suspected or culture-proven C-UTI with ENUC risk factors such as diabetes, immunosuppression, nephropathy, obstructive uropathy, urologic instrumentation and surgery [1–3].

Furthermore, in our experience, CT signs of clinically unsuspected C-UTIs are often detected incidentally on studies performed to investigate other conditions such as urolithiasis, renal colic, gynaecologic complaints or unspecific abdominal pain. When these abnormalities affect the scrotum, additional CD-US is indicated for diagnostic confirmation [10].

### Multidetector CT technique

Indwelling bladder catheters should be closed before performing CT, although in most patients with lower urinary tract dysfunction, bladder distension is poorly tolerated. A preliminary unenhanced acquisition is useful for detecting calcific urolithiasis, which may require specific treatment. Unless contraindicated by history of allergy or impaired renal function, administration of 110 to 130 ml of non-ionic low- or iso-osmolar iodinated contrast medium (CM) such as 350 mgI/mL iomeprol, 320 mgI/ml iodixanol, or 370 mgI/mL iopromide is generally beneficial [4, 6]. According to the European Society of Urogenital Radiology (ESUR) guidelines, special care in ensuring adequate hydration before and after CT is recommended to improve urinary tract opacification and to prevent CM nephrotoxicity in septic or dehydrated patients with limited urine output [11, 12].

In patients with clinically suspected or confirmed UTI, a single breath-hold nephrographic CT acquisition encompassing the abdomen, pelvis and perineum is obtained after intravenous CM injection. On our 64-slice CT scanner, the usual acquisition parameters include 80–140 KV according to body size and weight, automated tube current modulation, 0.75 s rotation time, and 64 × 0.625 mm collimation. Additional excretory phase imaging acquired 5–20 min after CM injection visualises the opacified urinary collecting systems, ureter and bladder. In contrast to classical multiphasic CT studies, modern split-bolus CT urography protocols provide combined corticomedullary, nephrographic and excretory imaging with a reduced effective radiation dose in a single volumetric acquisition. Multidetector CT studies tailored to the urinary tract should be routinely reconstructed along axial, coronal and sagittal planes, and may be reviewed interactively at the workstation and complemented by additional focused reconstructions such as coronal maximum-intensity projection (MIP) pyelographic images [13, 14].

CT is very appealing to clinicians and radiologists, since it provides a comprehensive anatomic and functional assessment of the entire urogenital tract, including information on renal perfusion and opacification of the urinary cavities. However, it should be used with caution, given its key intrinsic disadvantage of irradiation risk, particularly in young patients and women of reproductive age. With the above-described protocol, the estimated radiation exposure from each phase of acquisition generally falls in the range of 8 to 12 mGy. Strategies for dose reduction, such as automated tube current modulation or, if available, iterative reconstruction from raw CT data, are recommended [15].

### MRI role and technique

Albeit limited by lengthy examination time and the need for cooperation in acutely ill patients, MRI provides an excellent multiplanar visualisation of pelvic, genital and perineal structures, with superior contrast resolution and better tissue characterisation than CT. However, MRI is insensitive for the presence of gas and calcifications. Unless contraindicated by claustrophobia, early pregnancy, metallic foreign body in vital sites, cardiac pacemaker or other implanted devices, the use of MRI is increasingly appealing to avoid irradiation of the reproductive organs [16].

In the setting presented herein, MRI is recommended as a useful problem-solving modality to investigate disorders of the urinary bladder, prostate and seminal vesicles after inconclusive sonographic and CT findings, or as a first-line examination in younger patients [4–6].

MRI is arguably the best technique and therefore warranted for the provision of cross-sectional multiplanar evaluation of suspected or known periurethral and penile disorders [8, 17–19] and abnormalities affecting the perianal and perineal structures [20, 21] and the scrotum [22–24].

On current high-field-strength MRI scanners, pelvic studies are generally acquired with the use of phased-array coils; the patient lies supine, with a folded towel positioned between the legs to elevate the scrotum, and the penis secured at the midline hypogastrium. Most protocols rely on multiplanar high-resolution T2-weighted sequences, with short-tau inversion recovery (STIR) or spectral fat suppression in at least one plane. Additional static-fluid MR urography sequences may provide a panoramic view of the entire urinary collecting systems, without the use of intravenous contrast. Three-dimensional fat-suppressed gradient-echo T1-weighted sequences such as T1 high-resolution isotropic volume excitation (THRIVE), liver acquisition with volume acquisition (LAVA) or volumetric interpolated breath-hold examination (VIBE) are generally acquired before and after administration of a standard dose of gadolinium-based CM [5, 19, 25, 26].

## Cross-sectional imaging features of urinary bladder infections

### Acute infectious cystitis

**Fig. 1:**
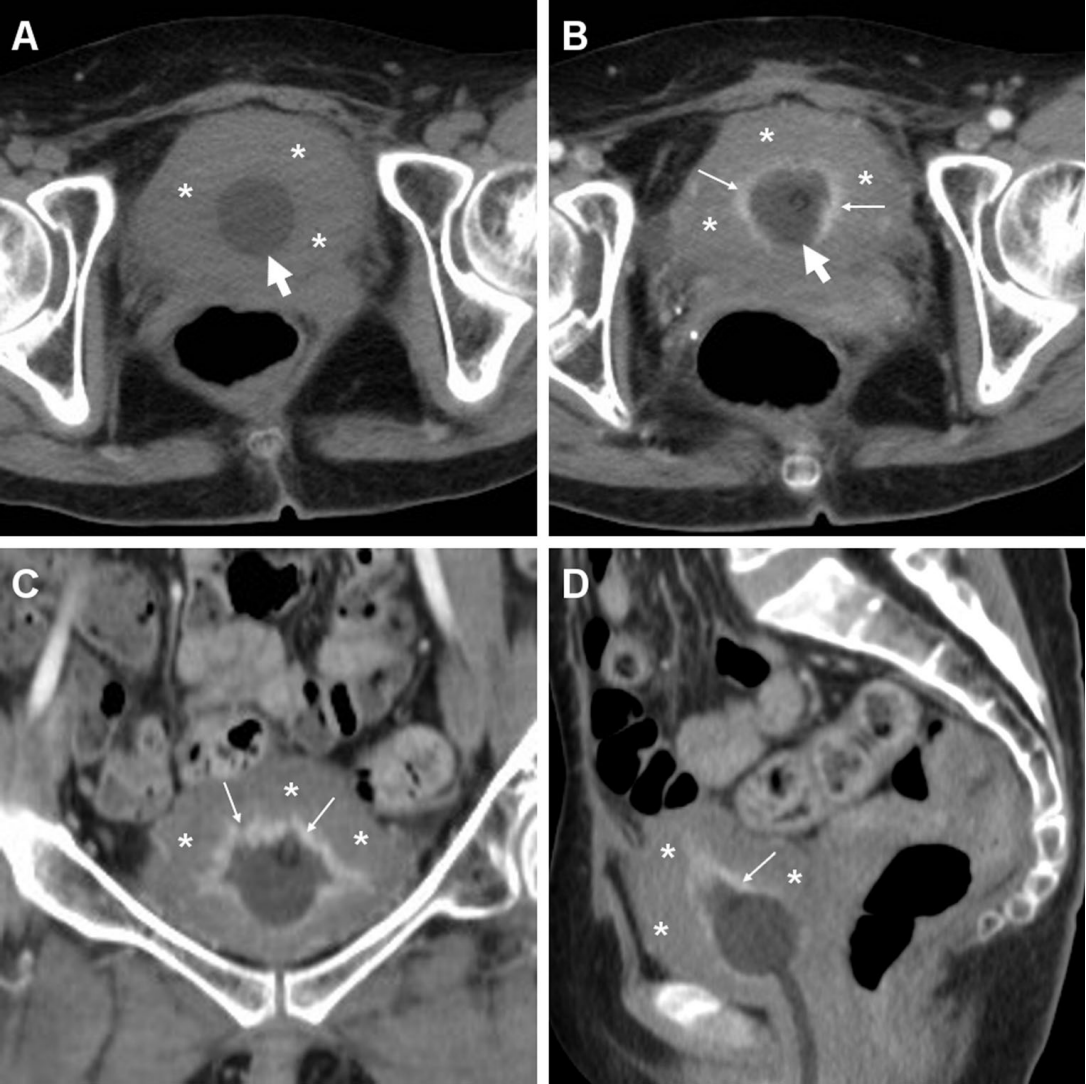
Active infectious cystitis in a 52-year-old woman with poorly controlled diabetes, dehydration, pelvic and flank pain, pyuria and elevated C-reactive protein (CRP). Unenhanced (**a**) and contrast-enhanced (**b**–**d**) multidetector CT images showed contracted urinary bladder with catheter (*thick arrows*), and marked circumferential mural thickening (*) with hypoenhancing oedematous wall and urothelial hyperenhancement (*thin arrows*). Urine cultures revealed polymicrobial infection including *Staphylococcus aureus* and multiresistant extended-spectrum beta-lactamase (ESBL)-positive *Escherichia coli*

**Fig. 2:**
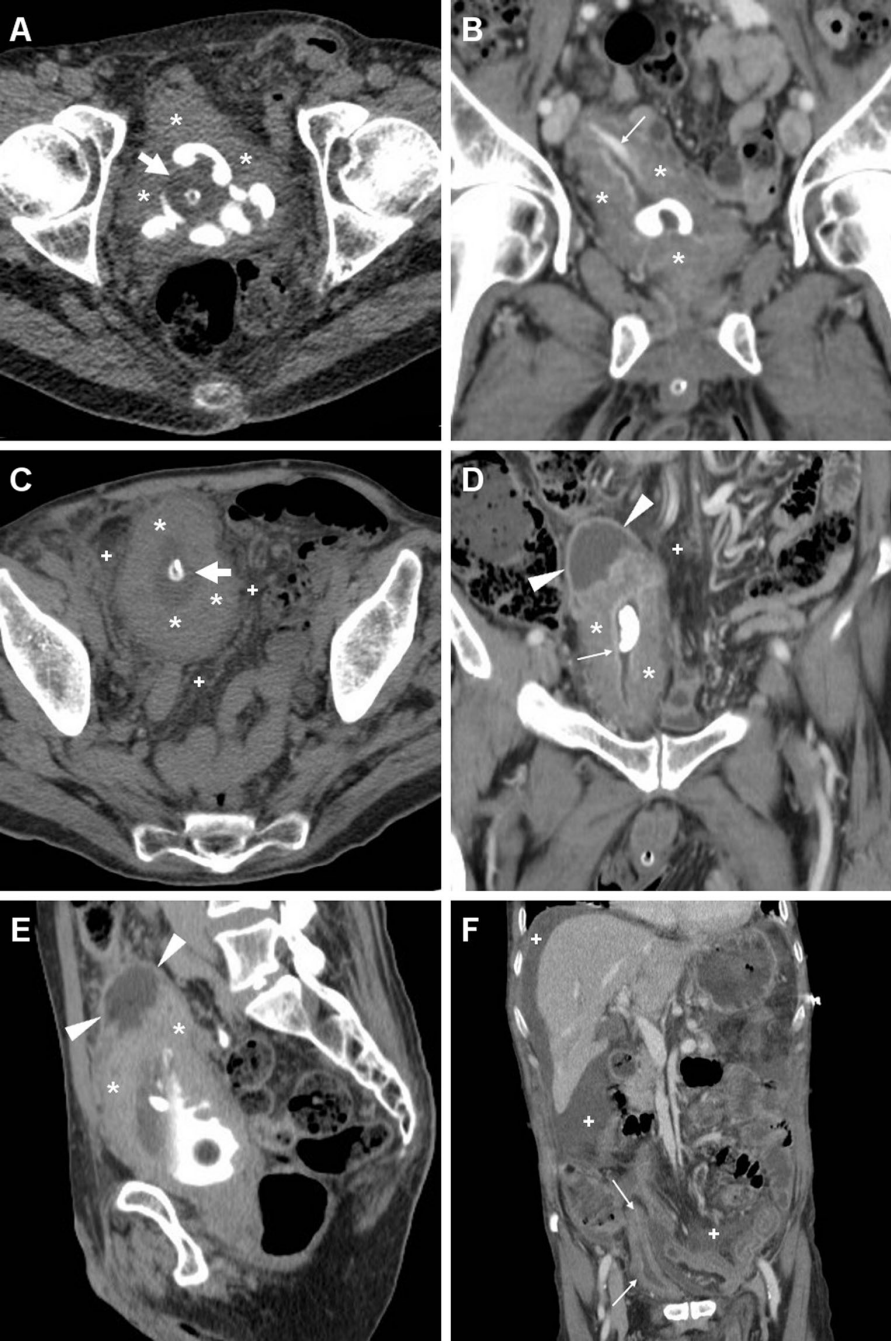
Polymicrobial urinary tract infection (UTI) complicated by mural bladder abscess in a 67-year-old man with a history of benign prostatic hyperplasia (BPH) and indwelling catheter (*thick arrows*). Four months earlier, CT (**a**) revealed contracted urinary bladder with calcific lithiasis, circumferential mural thickening (*) from detrusor hypertrophy, and urothelial hyperenhancement (*thin arrow in* **b**) consistent with active UTI. The current urgent CT (**c**–**e**) requested to investigate urosepsis showed increased mural thickening of the urinary bladder (*), with persistent urothelial enhancement (*thin arrows* in **d**), the appearance of inflammatory stranding of the perivesical fat planes (+), and the development of a sizeable (7.5×6×5.5 cm) collection attached to the bladder dome (*arrowheads*), with non-enhancing hypoattenuating (10–15 Hounsfield units, HU) content and enhancing peripheral rim. Cystoscopy confirmed severely inflamed bladder mucosa. Postoperative CT after surgical abscess drainage depicted normalised bladder wall (*thin arrows* in **e**) and appearance of ascites (*) [Partially reproduced from Open Access Ref. [27]]

**Fig. 3:**
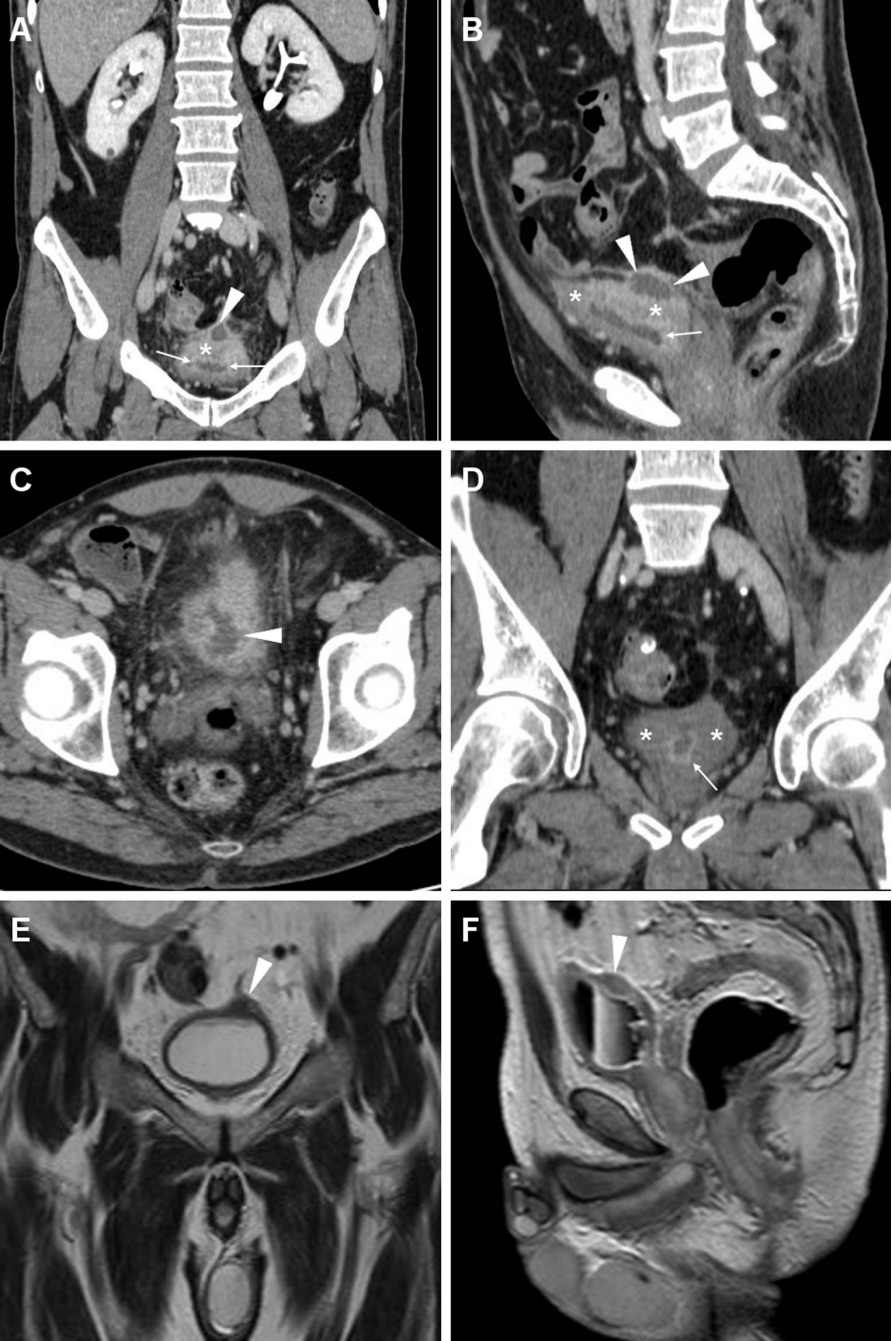
Mural bladder abscess in a 61-year-old man with recurrent UTIs and clinical and sonographic suspicion of bladder carcinoma. Multiplanar CT images (**a**–**d**) depicted a poorly distensible bladder with mural thickening (*), urothelial hyperenhancement (*thin arrows*) consistent with active UTI, and a fluid-like collection (*arrowheads*) with irregular peripheral enhancement along the posterolateral aspect. Urine cytology and cystoscopy excluded the presence of tumour. Six weeks later, repeated CT (**d**) after antibiotic treatment showed resolved abscess, persistent mural thickening (*) and infectious urothelial enhancement (*thin arrow*). Further follow-up with MRI including T2- (**e**) and post-gadolinium T1-weighted (**f**) sequences confirmed abscess disappearance (*arrowheads*)

The majority of patients experiencing lower C-UTIs have a scarcely distensible, thickened urinary bladder secondary to underlying chronic conditions such as detrusor hypertrophy and recurrent infections. Therefore, diffuse mural bladder thickening is commonly observed in patients undergoing CT for investigation of C-UTIs or urosepsis. However, in our experience, significant (over 1 cm) circumferential wall thickening may correspond to acute UTI, particularly when the muscular layer shows poor enhancement from intramural oedema (Figs. 1 and 2). Active infectious cystitis (AIC) is heralded by minimal, uniform thickening and hyperenhancement of the urothelium which lines the inner aspect of the urinary bladder (Figs. 1, 2 and 3). Similar to inflammatory enhancement described in acute pyelo-ureteritis, this appearance may be encountered in CT studies performed for different or unrelated indications, may be confirmed by performing slab maximum-intensity projection (MIP) reconstructions (Fig. 2b), and should be reported as consistent with AIC [4, 28].

**Fig. 4:**
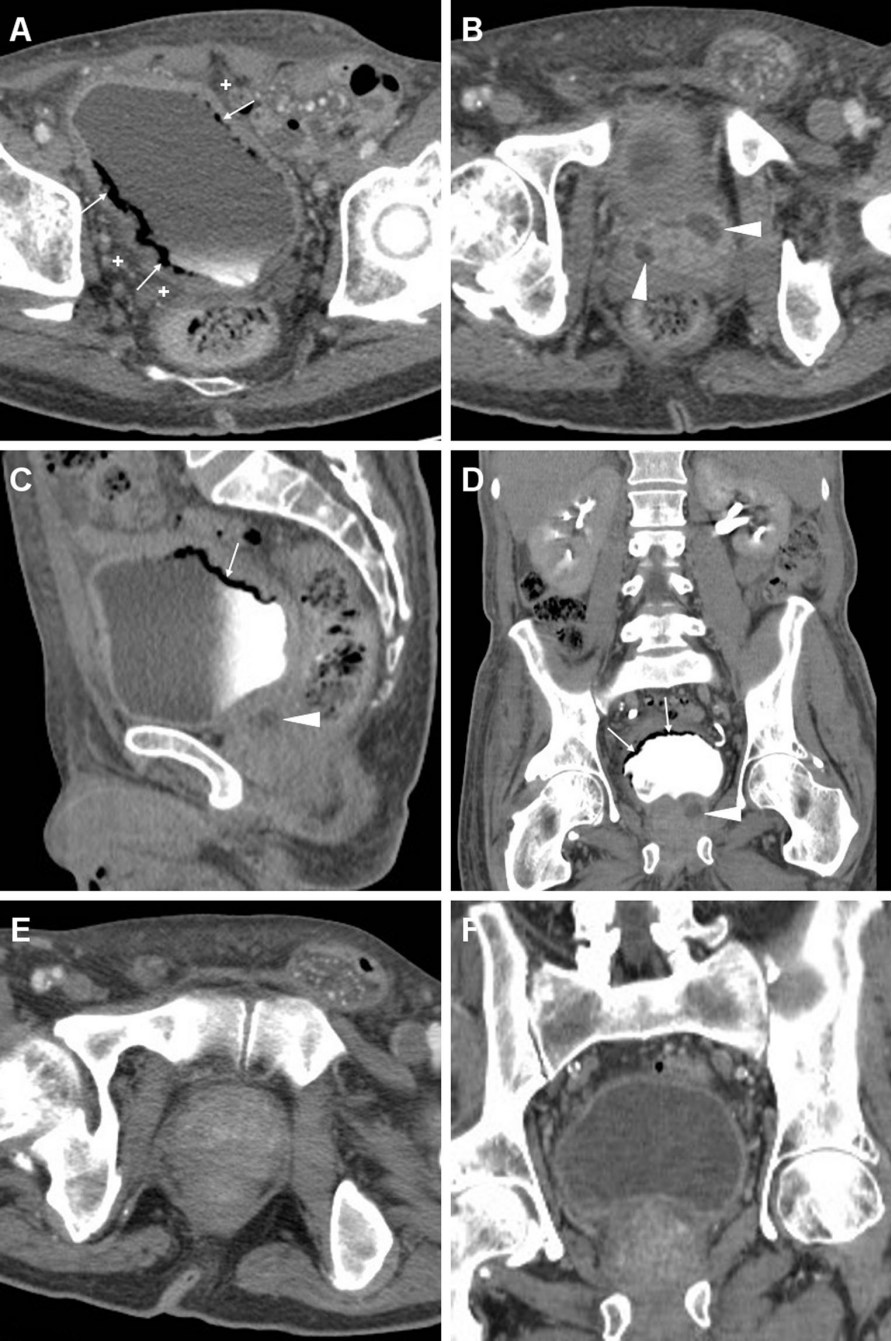
Emphysematous cystitis in a 69-year-old man with congestive heart failure, diabetes and chronic obstructive lung disease, suffering from urinary frequency and pain. CT urography (**a**–**d**) revealed distended urinary bladder with linear gas-attenuation changes (*thin arrows*) along the right lateral and upper posterior walls. Associated findings included inflammatory stranding (+) of the perivesical fat planes and a small bilobated fluid-like intraprostatic collection consistent with abscess (*arrowheads*). Follow-up CT (**e**, **f**) 3 months later revealed resolution of changes after prolonged antibiotic therapy

In addition, diagnosis of AIC is further suggested by the identification of inflammatory changes as “hazy” increased attenuation of the extraperitoneal perivesical fat planes (Figs. 2 and 4). Particularly frequent in diabetics, emphysematous cystitis is a form of C-UTI in which gas-forming micro-organisms lead to the formation of characteristic air-attenuation linear changes within the bladder wall (Fig. 4) [6].

**Fig. 5:**
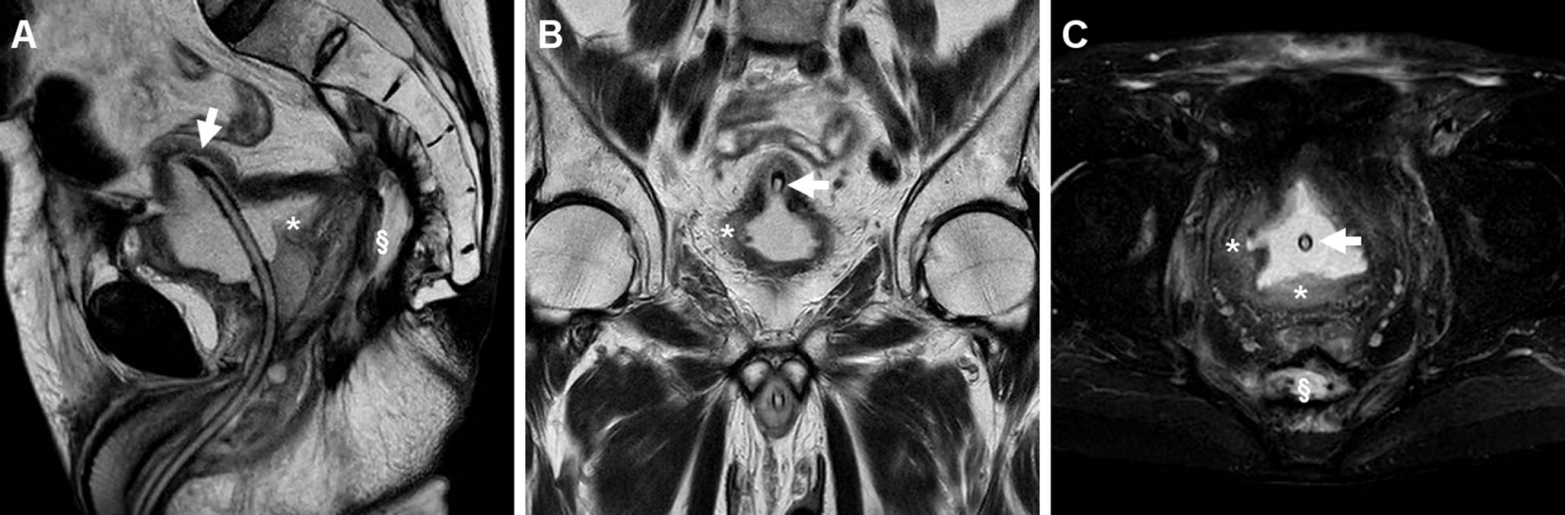
Acute infectious cystitis in 66-year-old bedridden man with several comorbidities and urosepsis. After inconclusive abdominopelvic CT (not shown), MRI showed contracted bladder with Foley catheter (*thick arrows*) and diffuse mural thickening with multifocal high-signal oedematous regions, best appreciated with fat saturation (**c**)

**Fig. 6:**
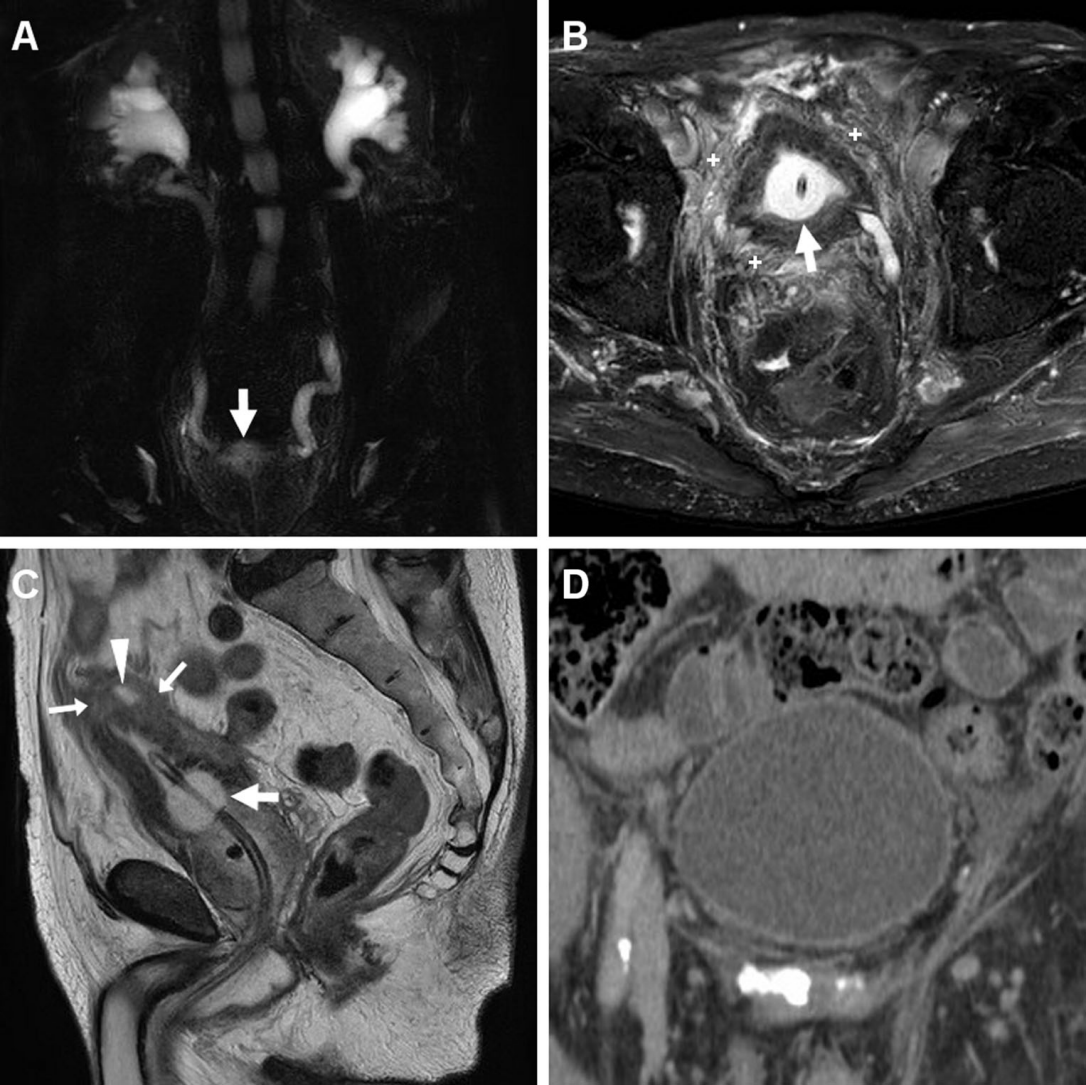
Acute infectious cystitis with mural bladder abscess in an 89-year-old man with acute urinary retention, fever, leukocytosis and impaired renal function. Unenhanced MRI including MR pyelographic (**a**) and axial fat-suppressed (**b**) images revealed bilateral hydronephrosis, contracted bladder and prominent inflammatory changes (+) in the surrounding extraperitoneal fat planes. Additionally, sagittal T2-weighted image (**c**) showed a focal thickening (*arrows*) at the bladder dome, with intramural fluid collection (*arrowhead*). Repeated CT (**d**) after medical treatment revealed the disappearance of abnormal mural changes

Compared to CT, MRI (Figs. 3, 5 and 6) allows superior assessment of the urinary bladder wall, which in normal conditions shows uniformly low T2-signal intensity (SI) corresponding to the detrusor muscle. At MRI, AIC is heralded by focal or diffuse mural oedema (Fig. 5) and inflammatory-type T2 hypersignal of the perivesical fat (Fig. 6), which is most appreciable with fat suppression techniques [4–6].

### Urinary bladder abscess

At times, AIC may be further complicated by the formation of a mural bladder abscess, which characteristically appears as an intramural or exophytic non-enhancing hypoattenuating (10–15 HU) collection with irregular peripheral enhancement, generally developing at the bladder dome (Figs. 2, 3 and 6). The lack of an appreciable communication with the bladder lumen allows differentiation of a mural abscess from an infected diverticulum. A correct diagnosis of bladder abscess is crucial, since long-term catheterisation plus drainage (either percutaneous or surgical) is required to relieve the septic focus [27, 29–31].

### Differential diagnosis of infectious bladder changes

**Fig. 7:**
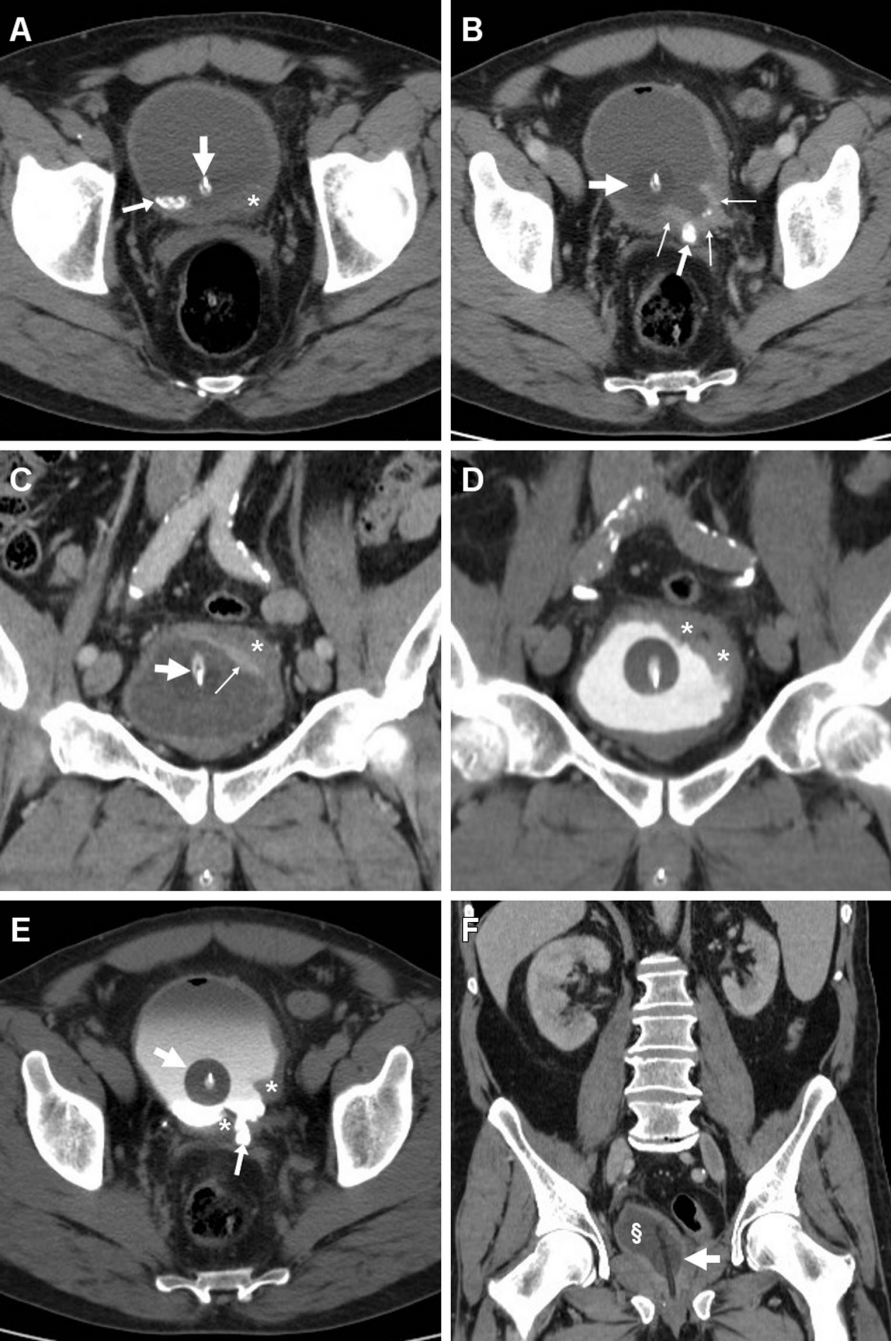
Muscle-invasive bladder carcinoma in a 54-year-old man with urolithiasis (*arrows*) and long-term bladder catheterisation (*thick arrows*). Unenhanced (**a**), portal (**b**, **c**) and excretory (**d**, **e**) phase CT images showed focal solid mural thickening (*) at the left posterolateral bladder wall, with an irregular configuration and positive contrast enhancement (*thin arrows*). Postoperative status after radical cystectomy (**f**) with orthotopic neobladder (§) is shown on follow-up CT (**f**)

The differential diagnosis of AIC includes bladder carcinoma and certain non-neoplastic disorders which manifest as an abnormal bladder wall with decreased distensibility and focal or diffuse mural thickening [32]. Largely employed as the initial investigation in patients with gross or recurrent microscopic haematuria, multidetector CT depicts transitional cell carcinoma as a uni- or multifocal, generally asymmetric wall thickening, which enhances mostly after a 60-s delay (Fig. 7). Tumour is suggested over infection when a soft-tissue density irregularity is detected at the interface between mural thickening and perivesical fat. Perivesical invasion is obvious when the neoplasia shows overt growth beyond the outer bladder wall contour. MRI is superior to CT in detecting tumour tissue disrupting the hypointense outer muscle layer and extending transmurally into the perivesical fat [33–35].

Nephrogenic adenoma (NA) and malacoplakia of the urinary bladder occur mostly in diabetic or immunocompromised individuals, and manifest with haematuria, proteinuria and signs of lower urinary dysfunction. The latter is a rare chronic granulomatous condition; the former results from long-term irritation from calculi, infection, injury or previous surgery causing urothelial metaplasia. Both entities show unspecific imaging features such as multifocal asymmetric thickening or intraluminal vegetations, frequently associated with hydronephrosis [32, 36].

**Fig. 8:**
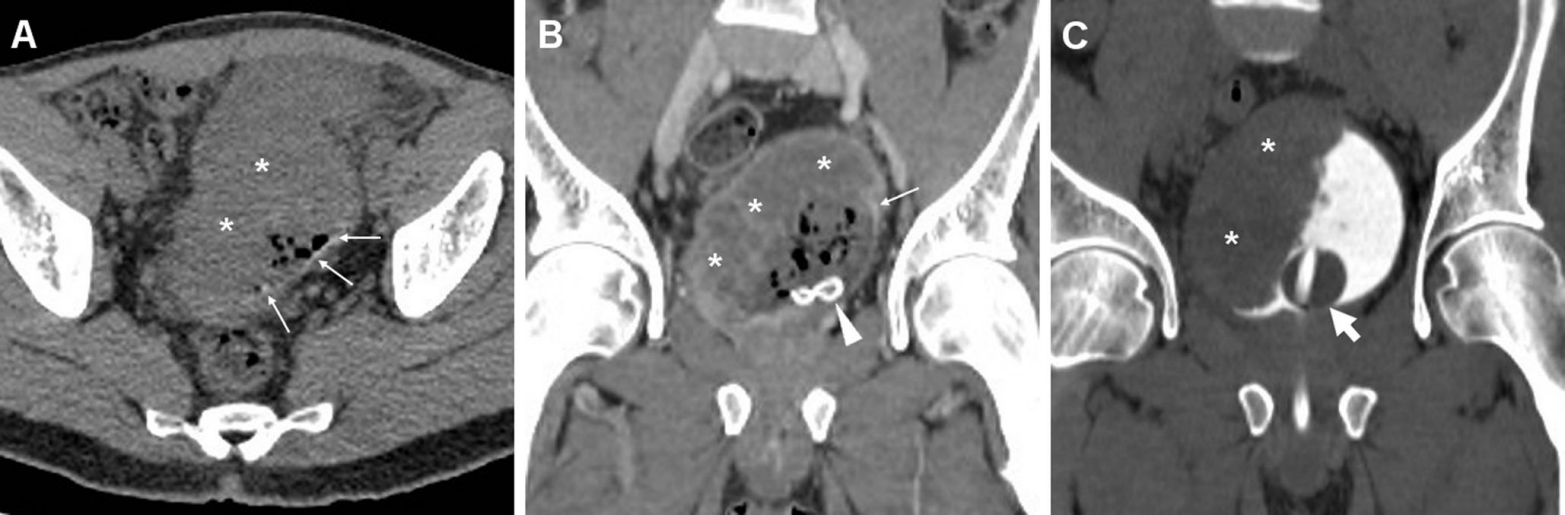
Schistosomiasis with superimposed squamocellular carcinoma in a 48 year-old Gambian man with pelvic pain and tenderness, dysuria and difficult urination. Unenhanced (**a**) and post-contrast (**b**) CT images showed marked asymmetric solid mural thickening (*), with poor enhancement along the anterior right lateral and superior bladder aspects. Additional findings included thin calcifications (*thin arrows*) along the left posterolateral bladder wall, and intraluminal stones (*arrowhead*). CT-cystography (**c**) with retrograde contrast filling and cystoscopy confirmed extensive bladder occupation by tumour (*) [Partially reproduced with permission from Ref.[37]]

Infections such as tuberculosis and schistosomiasis should be suspected in patients from endemic countries: the latter is still prevalent in areas of Africa and the Middle East, and is characterised by a contracted, thick-walled bladder with calcifications, and may be complicated by the development of squamocellular carcinoma (Fig. 8) [37–39].

Finally, other conditions which can mimic AIC and bladder carcinoma include rare inflammatory diseases such as cystitis cystica, cystitis glandularis, eosinophilic cystitis, chemotherapy (particularly with cyclophosphamide) and irradiation [32, 40, 41].

## Cross-sectional imaging features of infections of the prostate and seminal vesicles

### Prostatic infections and abscesses

With the widespread use of antibiotics, C-UTIs involving the prostate have become increasingly rare, and nowadays are primarily caused (almost two-thirds of cases) by *E. coli* infection. Acute bacterial prostatitis (ABP) is a serious infection which requires intensive parenteral antibiotics [1].

Prostatic abscesses (PAs) may result from unrecognised or inappropriately treated ABP, or may develop shortly after transrectal ultrasonography (TRUS)-guided biopsy. Most cases occur in the fifth and sixth decades of life, and the usual predisposing factors include diabetes, indwelling catheters, urological instrumentation and anatomical abnormalities. The non-specific clinical manifestations largely overlap with those of ABP, and include fever, dysuria or frequency, dull pelvic or perineal pain, rectal tenesmus, prostatic tenderness at digital rectal examination, and sometimes acute urinary retention. Laboratory findings indicate acute UTI, and increased serum prostate-specific antigen (PSA) is common but generally regresses with therapy. Urine cultures are often negative, and may become positive when the abscess opens into the urethra or bladder [42–44].

PAs may be detected by TRUS as single or multiple hypoechoic areas with thick walls, floating echogenic speckles in the cavity, and poorly defined periphery, with increased colour Doppler signals [45, 46].

**Fig. 9:**
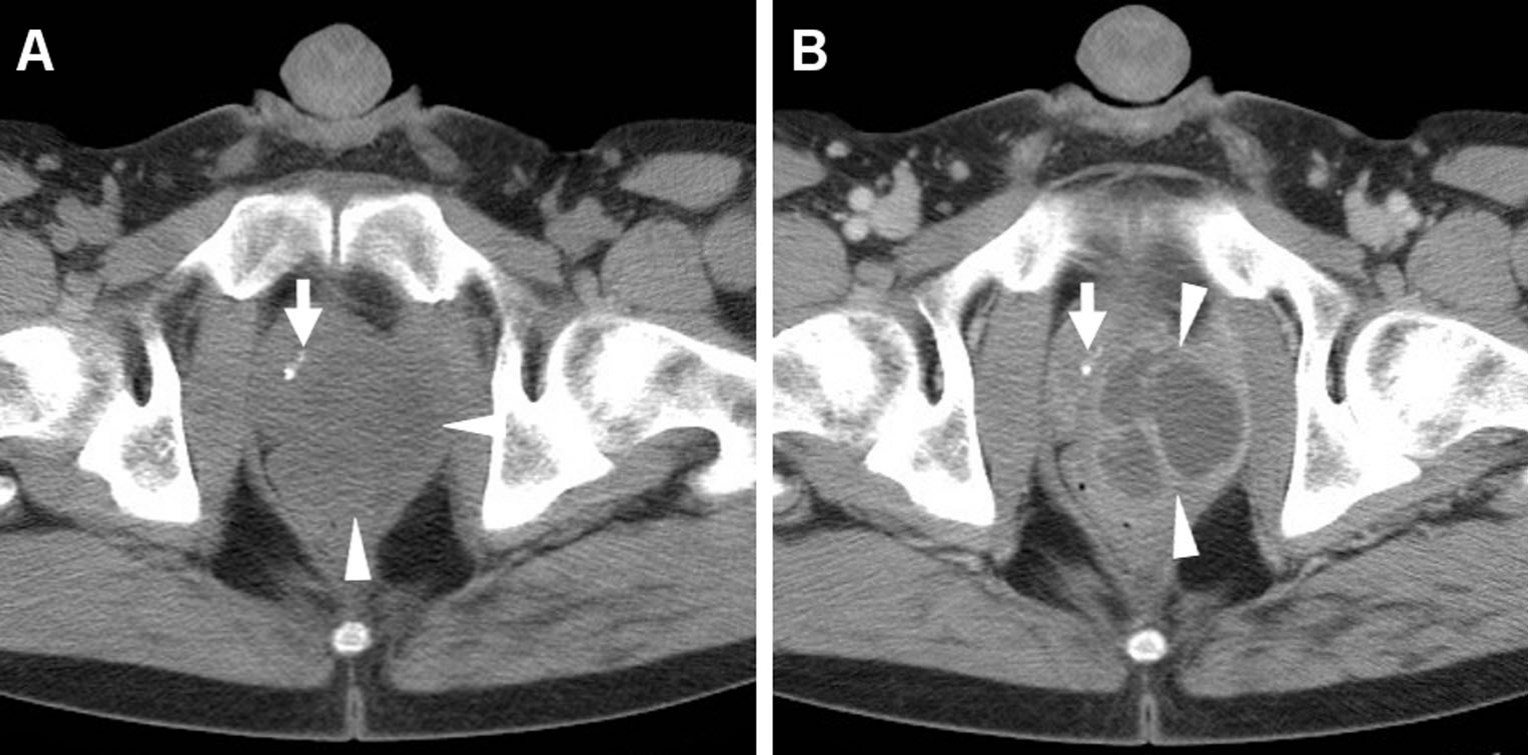
Prostatic abscess in a 48-year-old man with perineal pain and abnormally increased CRP. Axial unenhanced (**a**) and post-contrast (**b**) CT images showed mild asymmetric prostatic enlargement, occupied by a 4-cm septated fluid-like collection (*arrowheads*) with peripheral and septal enhancement. Note displacement of periurethral calcifications (*thick arrows*) from midline. Ultrasound-guided transperineal drainage confirmed *Escherichia coli* infection

**Fig. 10:**
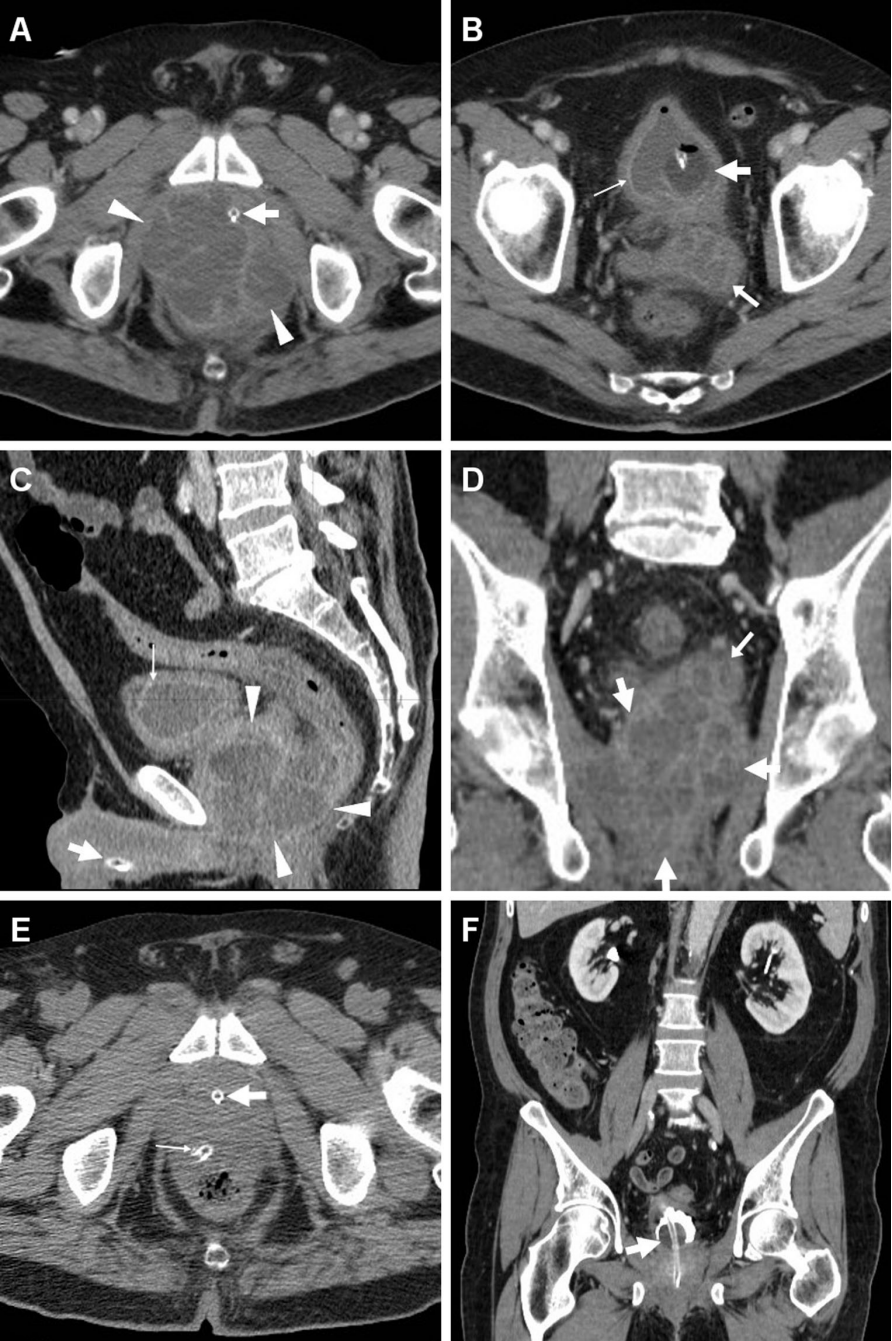
Large prostatic abscess from ESBL-positive *Escherichia coli* infection in a 61-year-old man with previous chemo- and radiotherapy for non-Hodgkin lymphoma, fever (38 °C), dysuria, pelvic pain and enlarged tender prostate at digital rectal examination. Multiplanar CT images (**a**–**d**) showed marked prostatic enlargement by confluent nonenhancing hypoattenuating (17–19 HU) regions, with peripheral and septal enhancement (*arrowheads*). The prostatic infection also involved the left seminal vesicle (*arrows* in **b**, **d**), displaced upwards of the urinary bladder, with mild circumferential mural thickening and mucosal hyperenhancement (*thin arrows*) consistent with UTI. After transperineal evacuation (**e**), follow-up CT urography (**f**) confirmed persistent resolution of the abscess [Partially reproduced from Open Access Ref. [47]]

**Fig. 11:**
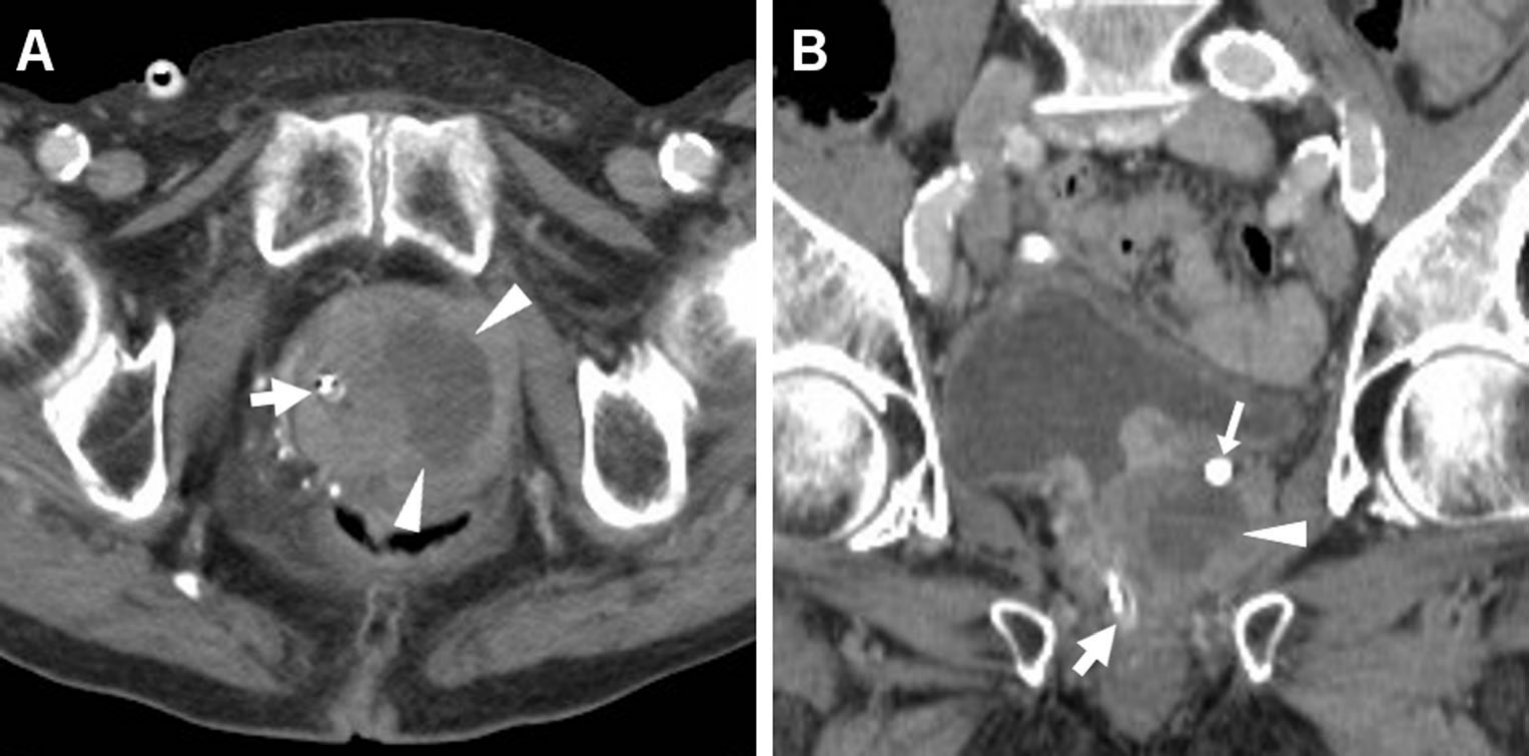
Prostate carcinoma with post-treatment regressive (necrotic) changes in an 86-year-old man with indwelling catheter (*thick arrows*). Axial (**a**) and coronal (**b**) post-contrast CT images depicted a 3×2 cm left-sided hypoenhancing region (*arrowheads*) causing enlargement of the ipsilateral prostate gland, initially interpreted as an abscess. Note residual brachytherapy seed indicated by arrow in **b**

Compared to TRUS, CT with multiplanar image reconstruction provides a more comprehensive visualisation of PAs. The usual appearance is single, septated or multiple fluid-like (−19 to 13 HU attenuation) collections, often with perceptible peripheral or septal enhancement, which cause more or less symmetric prostatic enlargement of variable entity (Figs. 4, 9 and 10). In addition, CT may depict signs of extraprostatic penetration (Fig. 10) in the prevesical space, rectum, perineum or ischiorectal fossa, and consistently allows monitoring of changes after medical or surgical treatment (Figs. 4 and 10). The differential diagnosis should consider prostate carcinoma, particularly with regressive changes after treatment (Fig. 11) [47–50].

Misdiagnosis or incomplete treatment of prostatic infections may result in serious morbidity, and the differentiation between ABP and PA is crucial, since it impacts management. Parenteral antibiotics plus transrectal ultrasound-guided or transperineal CT-guided drainage of abscesses are generally recommended as the initial approach in order to prevent sepsis. Alternatively, transurethral incision, deroofing or formal surgical evacuation may be required [1, 45, 46].

### Infections of the seminal vesicles

In the current antibiotic era, seminal vesicle abscesses (SVAs) are even more uncommon than PAs, and are frequently associated with ABP or epididymo-orchitis. SVAs may present with haemospermia or unspecific symptoms of UTI. However, both PAs and SVAs are often insidious and clinically unsuspected; therefore, a high index of suspicion is recommended when interpreting CT studies with the usual risk factors [42–44, 51–53].

**Fig. 12:**
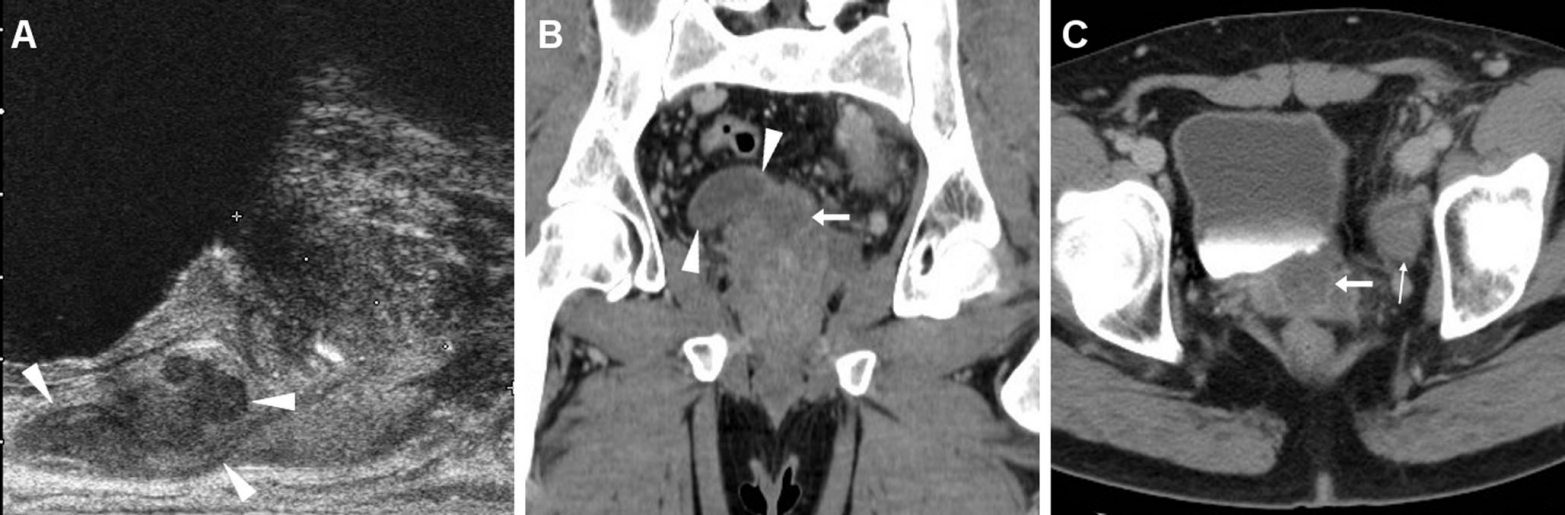
Chronic seminal vesicle infection in a 50-year-old HIV-positive man with haemospermia, appearing as hypoechoic enlargement (*arrowheads* in **a**) at transrectal ultrasound. CT urography (**b**, **c**) depicted a corresponding non-enhancing hypoattenuating “sac-like” structure with loss of normal septation (*arrowheads* in **b**), plus a focal hypoenhancing region at the prostatic base (*arrows*) and associated ipsilateral lymphadenopathy (*thin arrow*)

**Fig. 13:**
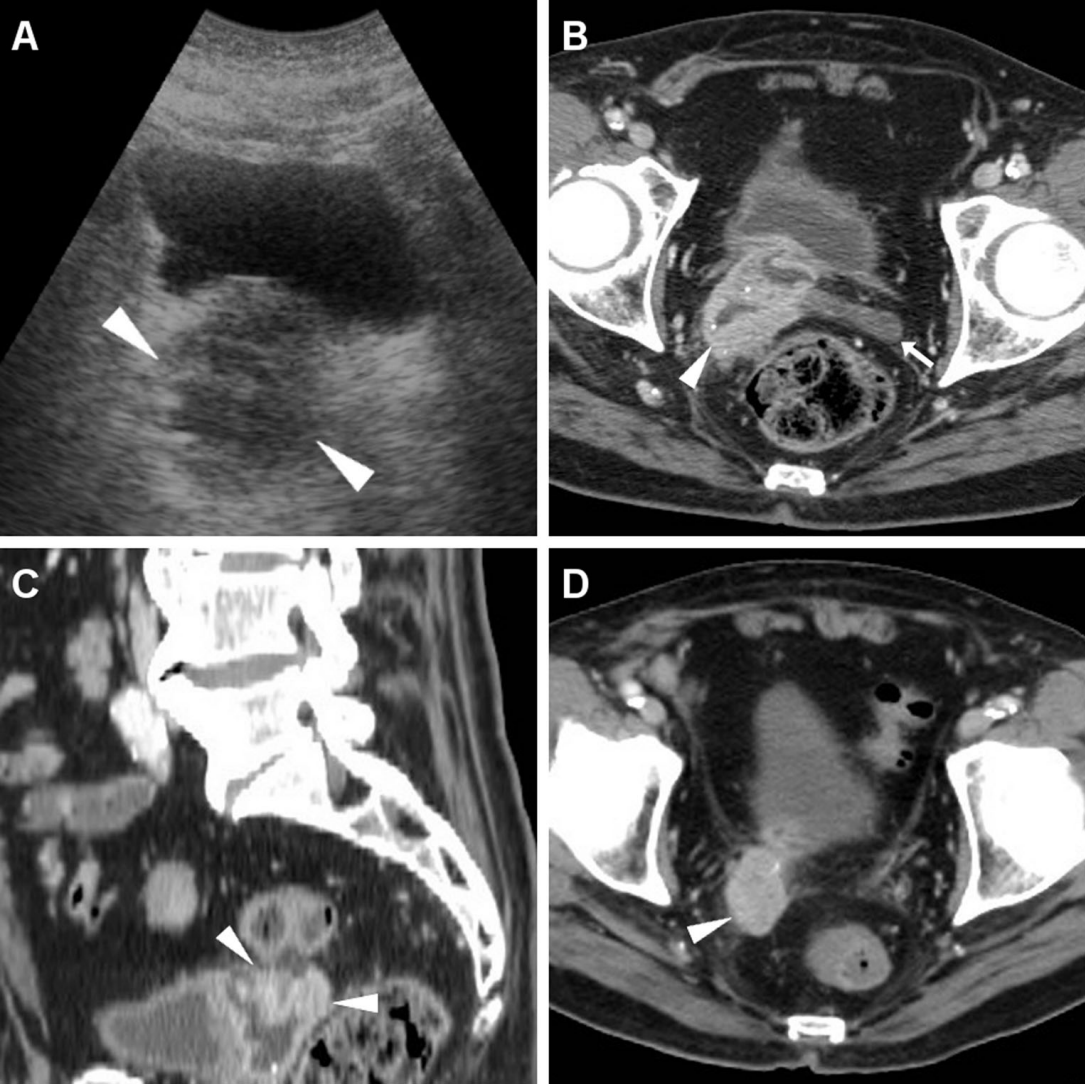
Seminal vesicle abscess in 74-year-old man with recurrent UTIs, suffering from malaise, persistent fever, pelvic tenderness and dysuria. Transabdominal ultrasound (**a**) revealed right paramedian inhomogeneous hypo-anechoic multiseptated mass (*arrowhead*), exerting compression on the urinary bladder. CT (**b**, **c**) confirmed markedly enlarged right seminal vesicle (*arrowheads*) with thick, strongly enhancing walls and septa, speckled calcifications, and internal liquefied areas. The abscess partially regressed, with disappearance of the mass effect and liquefied portions at follow-up CT (**d**), after intensive antibiotic treatment. Serum prostate-specific agent (PSA) normalised from 10 to 5 ng/mL over 2 months [Partially reproduced with permission from Ref. [54]]

Transabdominal or transrectal ultrasound may detect SVAs (Figs. 12 and 13) as hypo-anechoic masses [53, 55]. At cross-sectional imaging, the normal seminal vesicles appear as paired, finely septated fluid-containing structures, measuring approximately 3×1.5 cm in adults, and may be moderately asymmetric in size and tend to shrink with advancing age. Their content is normally homogeneous, with fluid-like CT attenuation and low T1- and fluid-like T2-hyperintense MRI signal with enhancing walls and septa after intravenous contrast [5, 52].

Cross-sectional CT imaging depicts SVAs as uni- or bilateral gland enlargement with thick irregular enhancing wall, internal hypoattenuating regions and adjacent fat inflammatory changes (Figs. 12 and 13). Imaging features of bladder and prostatic infection are frequently associated [52, 54, 56].

The reported MRI findings of subacute and chronic seminal vesicle infections include fluid-like septated collections, with more or less thick and marked peripheral enhancement [57, 58]. The differential diagnosis of seminal vesicle infections encompasses congenital cysts, acquired cystic dilatation, chronic tuberculosis, benign (cystadenoma, leiomyoma, teratoma) and malignant tumours, and metastases from prostate, bladder or rectal cancers [5, 52].

Although a conservative therapeutic approach is increasingly adopted, SVAs often require percutaneous transvesical drainage, transrectal aspiration or surgical incision [43, 44, 59].

## Cross-sectional imaging features of infections of the urethra, perineum and scrotum

### Urethritis

Spread through sexual contact, urethritis from *Neisseria gonorrhoeae* and *Chlamydia trachomatis* is favoured by promiscuity and low socioeconomic status. Alternatively, urethral infection increasingly results from intermittent or permanent catheterisation or from urologic instrumentation. Symptoms include mucopurulent discharge, alguria, dysuria and urethral pruritus [1, 7].

Acute urethritis is generally diagnosed on the basis of clinical and laboratory findings, but imaging may be required to exclude complications. In the past, conventional CM-enhanced radiographic studies were the primary modalities for imaging of the male urethra, particularly for assessment of traumatic injuries, strictures, and abscesses draining into the urethra. However, retrograde urethrography and voiding cystourethrography were unable to assess the periurethral structures [17, 60, 61].

**Fig. 14:**
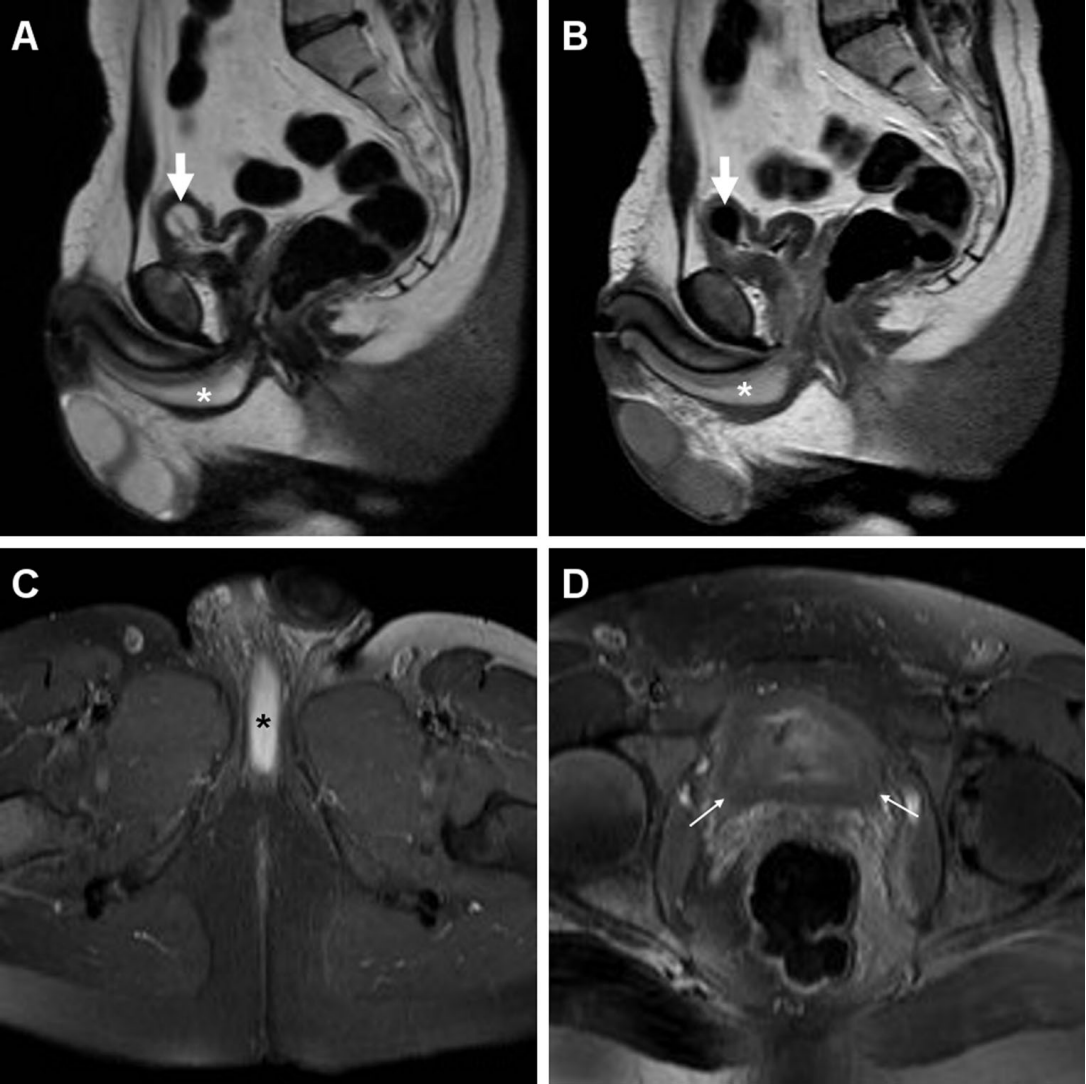
Acute uncomplicated urethritis in a 30-year-old man with neurogenic bladder treated by intermittent self-catheterisation. Physical examination revealed induration and tenderness of the corpus spongiosum and purulent urethral secretions. Unenhanced T2-weighted MRI images (**a**) revealed a diffuse, uniform hypersignal in the corpus spongiosum (*) with corresponding intense homogeneous enhancement on post-gadolinium T1- weighted sequences (**b**, **c**). The infection did not appear to interrupt the tunica dartos or Buck’s fascia, and did not involve the corpora cavernosa, scrotum or ischioanal spaces. Note Foley catheter in place (*thick arrows*). The patient successfully recovered with temporary suprapubic catheter and intravenous and topical antibiotics [Partially reproduced with permission from Ref. [62]]

Ultrasound of the penis may demonstrate a periurethral abscess, but is generally cumbersome because of inflammatory swelling and tenderness of the penile and perineal structures. The use of MRI can effectively visualise abnormalities of the periurethral structures [19]. In our experience, acute urethritis appears as diffuse thickening of the urethra and periurethral tissues, with intermediate to high signal intensity on T2-weighted images and intense contrast enhancement (Fig. 14) [5, 62].

### Perineal abscesses from lower urinary tract infection

Urethritis may be further complicated by a periurethral abscess through infection of Littré's glands. Since the penile tunica albuginea prevents the dorsal spread of infection, abscesses tends to track ventrally along the corpus spongiosum. When Buck's fascia is perforated, the process leads to fasciitis and gangrenous necrosis of the subcutaneous tissue [1].

**Fig. 15:**
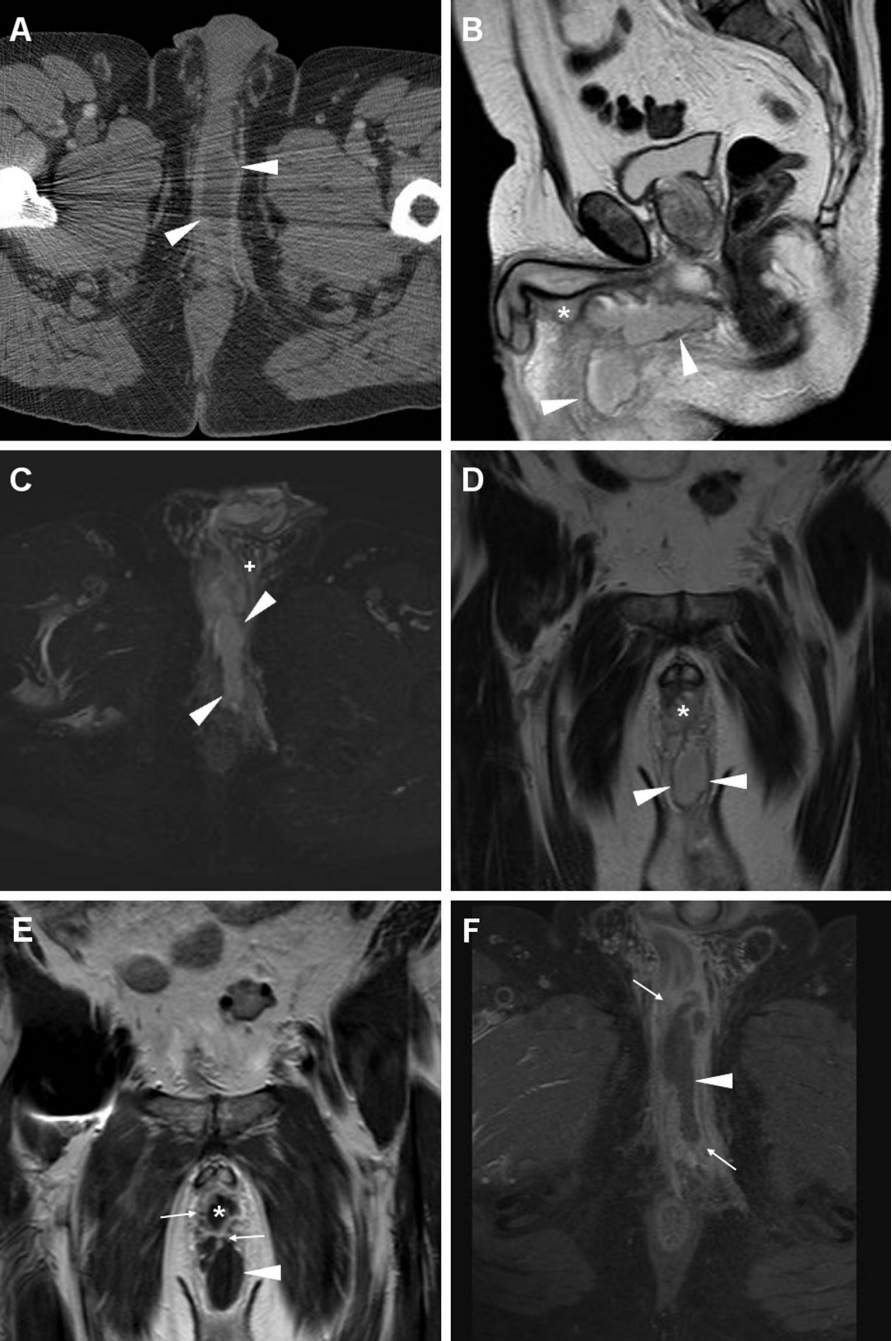
Urethral infection complicated by penile and perineal abscess in a 53-year-old man with tender, inflamed perineal swelling despite antibiotics. Infection was initially detected at contrast-enhanced CT (**a**) as an elongated midline abscess with peripheral enhancement (*arrowheads*) and internal fluid. MRI showed corresponding inhomogeneous fluid-like content on T2-weighted sequences (**b**–**d**), with surrounding inflammatory stranding (+) and strong contrast enhancement in the abscess walls (*arrowheads* in **e**, **f**). The infected corpus spongiosum (*) showed similar signal features. Surgical evacuation was required to relieve the abscess

MRI visualises perineal and penile abscesses as fluid- or pus-filled cavities with enhancing periphery, in communication with the urethra (Fig. 15), and may clearly reveal involvement of the corpora cavernosa and fibrous tunicae. A urethral diverticulum, most commonly located in the distal urethra, may mimic the appearance of an abscess. Treatment requires antibiotics, suprapubic urinary drainage and surgical evacuation [5, 24, 62, 63].

### Funiculitis and epididymitis

Colour Doppler ultrasound (CD-US) remains the primary modality for investigating abnormalities of the testis and epididymis, particularly to differentiate torsion from inflammatory conditions [4, 8, 9].

Acute epididymitis is almost always unilateral and has a bimodal distribution, with the majority of cases occurring between 16–30 and 51–70 years of age. Whereas in the former age group, infection is commonly caused by *Chlamydia trachomatis* or *Neisseria gonorrhoeae* and is associated with sexual activity, in individuals at an advanced age or immunosuppressed individuals, it results from C-UTI by common aerobic urinary pathogens such as *E. coli* [1, 64].

Clinical manifestations of epididymo-orchitis include scrotal pain, sometimes radiating to the groin or lower abdomen, fever and other symptoms of UTI, variable scrotal swelling and tenderness at physical examination. Laboratory tests, urethral swab and cultures help to direct antibiotic therapy, which cures infection, relieves symptoms and prevents complications and transmission [1, 64].

**Fig. 16:**
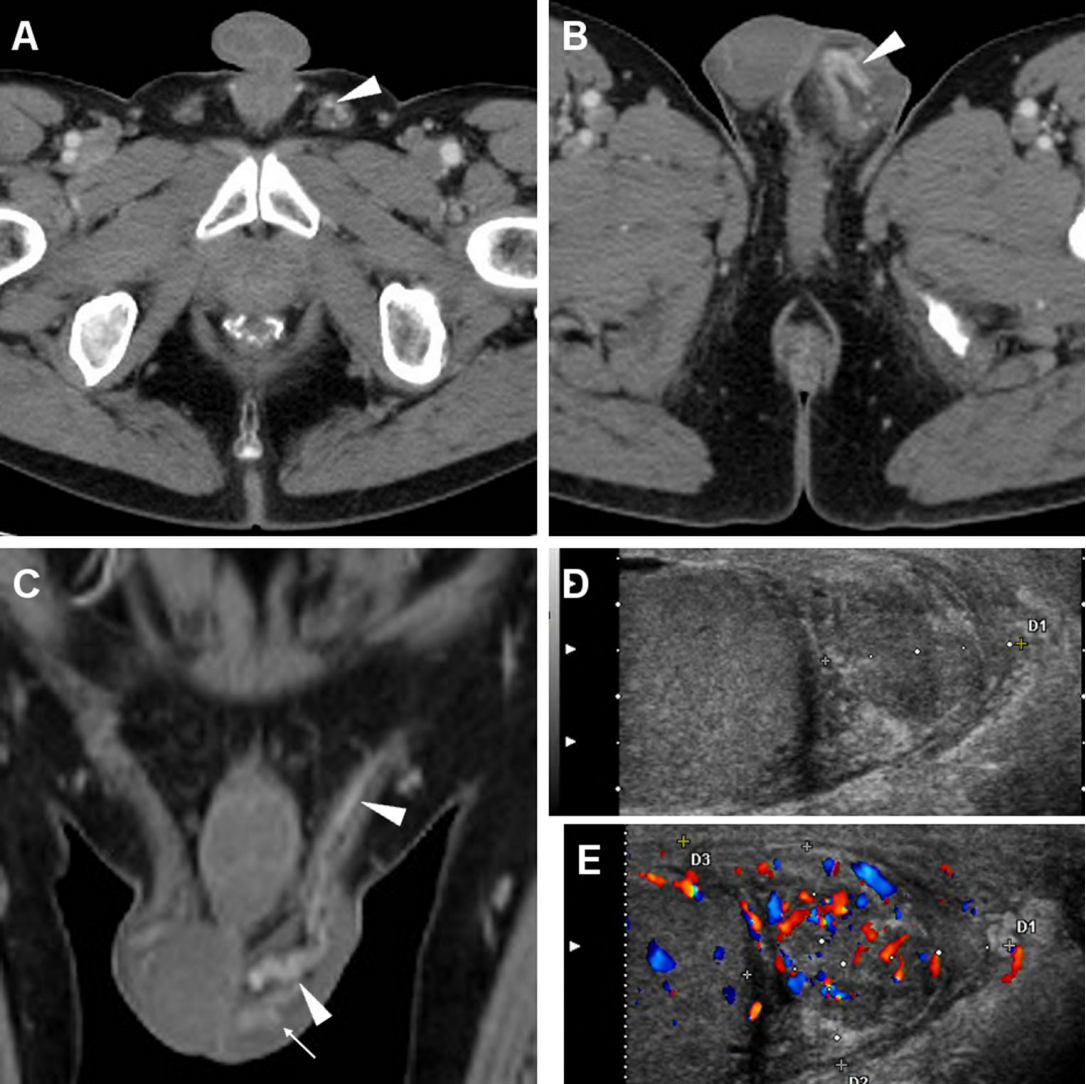
Unsuspected funiculitis and acute epididymitis in 53-year-old man with history of urolithiasis, fever, and left-sided abdominal pain radiating to the ipsilateral flank and groin. CT (**a**–**c**) did not confirm clinical suspicion of acute pyelonephritis. Subtle thickening and vascular engorgement (*arrowheads*) were noted along the left spermatic cord, plus faint hyperenhancement of the ipsilateral epididymal head (*thin arrow* in **c**). Additional colour Doppler ultrasound (CD-US) confirmed inhomogeneously hypoechoic (**e**), hypervascularised (**f**) epididymal head (calipers). Antibiotics effectively treated *Escherichia coli* infection [Partially reproduced with permission from Ref. [10]]

**Fig. 17:**
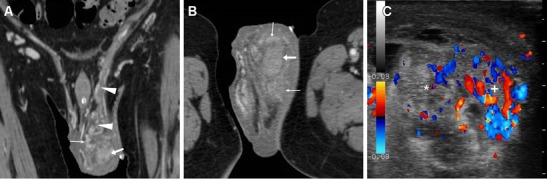
Acute epididymo-orchitis in a 47-year-old Sri Lankan man with multiple myeloma on bortezomib therapy, suffering from fever and acute scrotal pain, tenderness and induration. Contrast-enhanced CT (**a**, **b**) performed to investigate impending urosepsis depicted a thickened engorged left spermatic cord, with inhomogeneous vascularisation of the ipsilateral epididymis (*thin arrows*) and testis (*arrows*). Note catheter (in **a**), thickened and increased oedematous attenuation of the scrotal skin and external tunicae. CD-US (**c**) revealed hypervascularisation of the epididymis (+). Unresponsive to antibiotics, *Klebsiella pneumoniae* ultimately required orchiectomy

When CD-US is not the initial examination, careful scrutiny of CT studies may reveal spermatic cord asymmetry with variable thickening and increased enhancement of vessels on the affected side: this rather subtle finding reflects infectious hyperemia and is strongly associated with ipsilateral infectious funiculitis, epididymitis and/or orchitis; hypervascularity of the epididymis may also be observed at CT (Figs. 16 and 17). Alternatively, spermatic vascular engorgement may reflect the presence of a testicular tumour [10, 65].

**Fig. 18:**
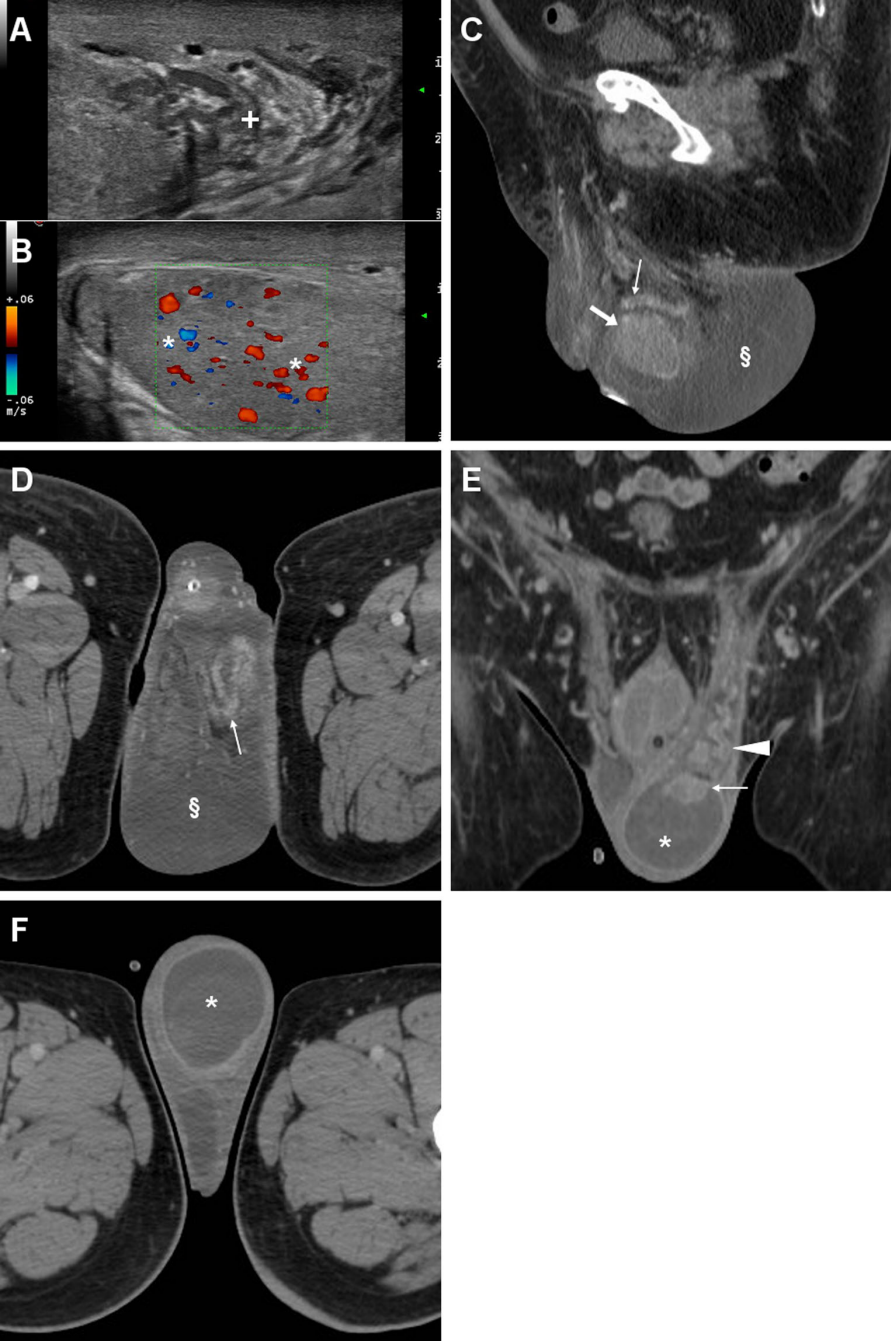
Surgically confirmed epididymo-orchitis with pyocele in a 72-year-old diabetic man with haematuria and enlarged left scrotum, history of transurethral resection of bladder carcinoma and bladder neck stricture treated by long-term catheterisation. Ultrasound revealed ipsilateral enlarged inhomogeneous epididymal head (+ in **a**) and hypervascularised testis (* in **b**). After unsuccessful antibiotic therapy, contrast-enhanced CT (**c**, **d**) showed hyperaemic left epididymis (*thin arrows*) and testis (*arrows*) compared to contralateral structures, and development of posterior scrotal collection (§). Another surgically proven case of testicular abscess and necrosis in a 59-year-old man with epididymo-orchitis unresponsive to medical therapy: post-contrast CT (**e**, **f**) revealed vascular engorgement along the left spermatic cord (*arrowhead*), faintly enhanced epididymal head (*thin arrow* in **e**), and ipsilateral scrotum occupied by fluid-like collection (*) with thin peripheral enhancing rim

Similarly, MRI may depict an enlarged epididymis with increased or heterogeneous T2 signal intensity and engorged vessels [5, 22, 24]. The diagnosis of epididymitis is easily confirmed by CD-US, with the usual features including segmental or global enlargement, inhomogeneously hypoechoic and hypervascularised compared to the testis, commonly associated with thickening of scrotal tunicae or hydrocele (Figs. 16, 17 and 18) [8, 9, 61].

### Orchitis and scrotal abscesses

Contiguous infectious involvement of the testis (orchitis) is rather uncommon compared to epididymitis, and generally has similar or more severe clinical and laboratory manifestations [1, 64].

CD-US hypervascularity is the key sonographic finding which allows differentiation of acute orchitis from torsion and infarction [8, 9].

At CT, the normal testes are symmetric and hypoattenuating, and are poorly differentiated by thickened scrotal tunicae and hydrocele. On cross-sectional imaging studies, acute orchitis is suggested by asymmetry with enlargement and increased contrast enhancement of the affected testis compared to the contralateral structure; signs of funiculitis and epididymitis are generally associated (Figs. 17 and 18). In normal conditions, at MRI the testes appear homogeneous, T1-isointense to muscle and T2-hyperintense, and the albuginea and mediastinum testis are identifiable as low-signal bands. Testicular inflammation is better demonstrated by MRI as decreased T1 and increased T2 signal intensity compared with the normal testis, with either intense homogeneous enhancement or the characteristic “tiger skin” post-contrast pattern corresponding to preserved septa. Focal or diffuse orchitis may be challenging to differentiate from testicular tumours, which generally show a mass effect and solid-type CT attenuation and MRI signal features, and are not associated with clinical and biochemical signs of infection [5, 22–24].

Untreated epididymo-orchitis may be further complicated by testicular necrosis and/or development of a scrotal abscess or pyocele, which requires surgical treatment [1, 64]. Abscesses may be sonographically appreciated as an ill-defined lesion with low echogenicity and absent internal flow signals [4, 8, 9]. CT and MRI features of pyocele include complex, heterogeneous fluid collections, surrounded by an enhancing periphery (Fig. 18) or by hyperaemic inflamed surrounding parenchyma [22–24].

### Differential diagnosis of perineal and genital infections

Fournier’s gangrene (FG) is a rare, life-threatening necrotizing polymicrobial infection of the perineal, perianal and genital structure, commonly occurring in diabetics, with a striking male predominance (male-to-female ratio, 10:1). FG may further complicate a lower UTI, or originate from different infections such as colorectal infections, anal fistulas or pressure ulceration. Clinical presentation includes local pain, swollen oedematous or gangrenous overlying skin, sometimes with appreciable crepitus, and progressive hyperthermia. FG represents a surgical emergency: aggressive surgical debridement and broad-spectrum antibiotics are required to prevent a fatal outcome [66, 67].

**Fig. 19:**
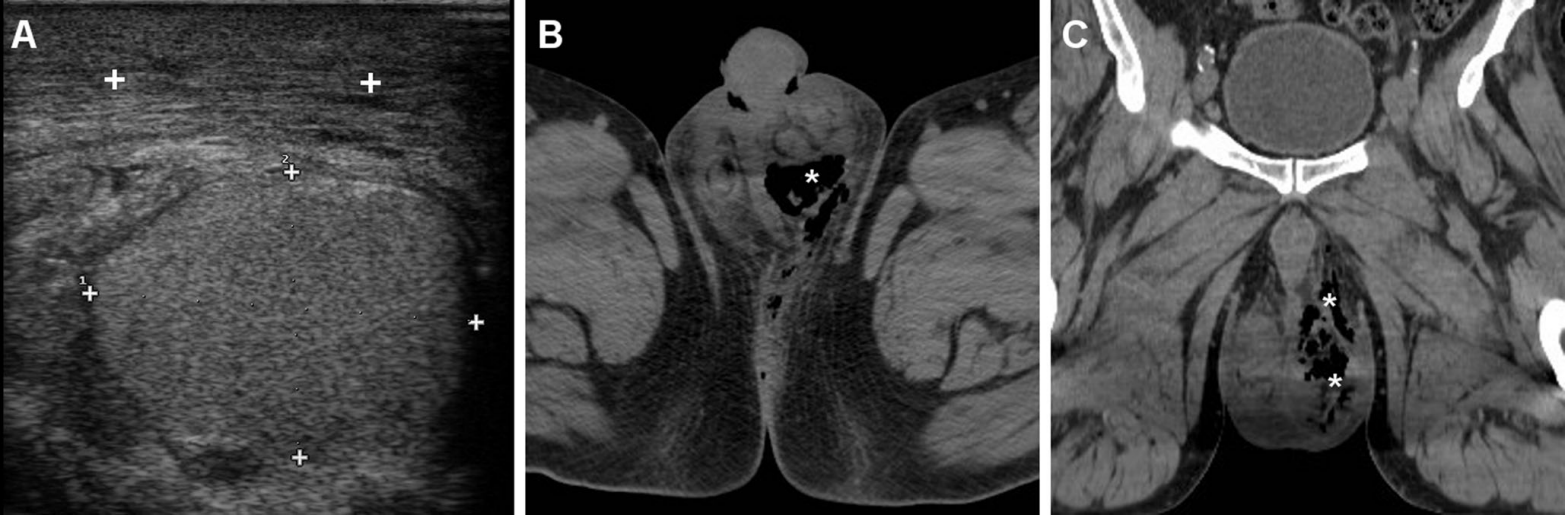
Clinically unsuspected, surgically treated Fournier’s gangrene in a 60-year-old man with diabetes, recurrent UTIs and scrotal swelling. Initial ultrasound (**a**) depicted prominent inflammatory thickening of the superficial tissues, without hydrocele, and signs of acute orchitis (testis is indicated by calipers). Unenhanced CT (**b**, **c**) revealed the presence of intrascrotal gas collections (*)

**Fig. 20:**
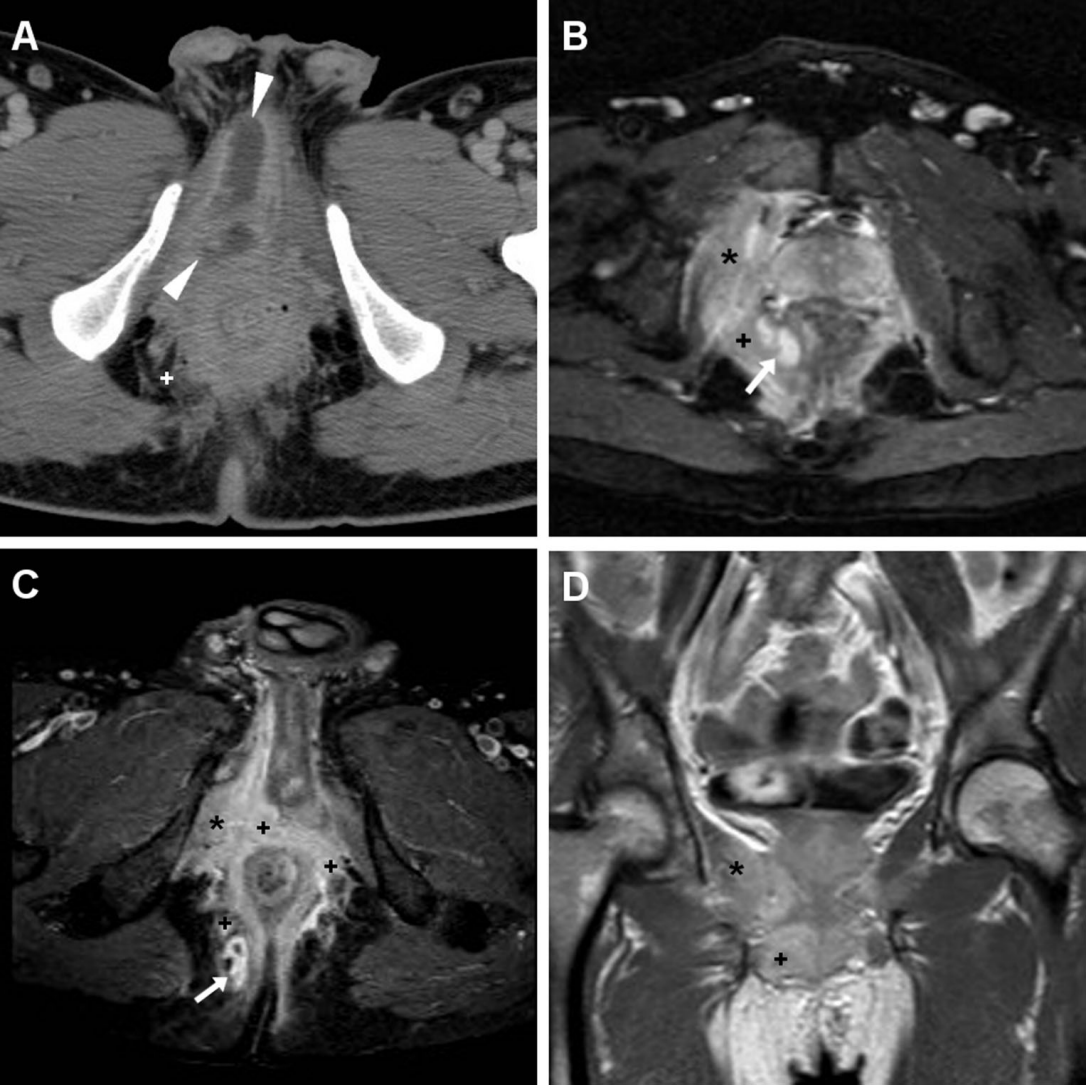
Extensive cryptogenic perianal inflammation in a 56-year old diabetic man with fever. Axial post-contrast CT image (**a**) revealed perineal abscess (*arrowheads*) very similar to that depicted in Fig. 15. Additional MRI including axial STIR (**b**), post-gadolinium axial fat-suppressed (**c**) and coronal (**d**) T1-weighted images showed extensive inflammatory signal abnormalities and hyperenhancement (+) surrounding the anus, involving the right sphincter complex and obturator internus (*) muscles, and extending to the ischioanal fossa. Tiny abscess collections (*arrows* in **b** and **c**) were present. Topography of infection, sparing of prostate and corpora cavernosa, and clinical examination were inconsistent with complicated UTI

CT is by far the best modality for imaging of FG; it quickly and comprehensively visualises the extent of FG involvement. CT features include subcutaneous fat stranding at the involved areas, fascial thickening, superficial or deep fluid and air-attenuation collections. Subcutaneous emphysema produced by anaerobic bacteria is the hallmark of FG and is present in approximately 90 % of cases (Fig. 19). Furthermore, CT can define the starting point of the infectious process, thereby allowing differentiation of complicated perineal infections from urinary versus an alternative source, particularly cryptogenic perianal sepsis (Fig. 20) [66–68].

Another uncommon differential diagnosis of perineal and scrotal infections is hidradenitis suppurativa (HS), an inflammatory disease of the skin and subcutaneous tissues with unclear pathogenesis and chronic progressive course. Mostly encountered in males and black people with poor hygiene, HS presents with tender subcutaneous nodules, which progress to form painful, deep dermal abscesses, sinus tracts, and eventually ulceration and fibrosis. Recurrence is common despite surgical excision [69, 70].

**Fig. 21:**
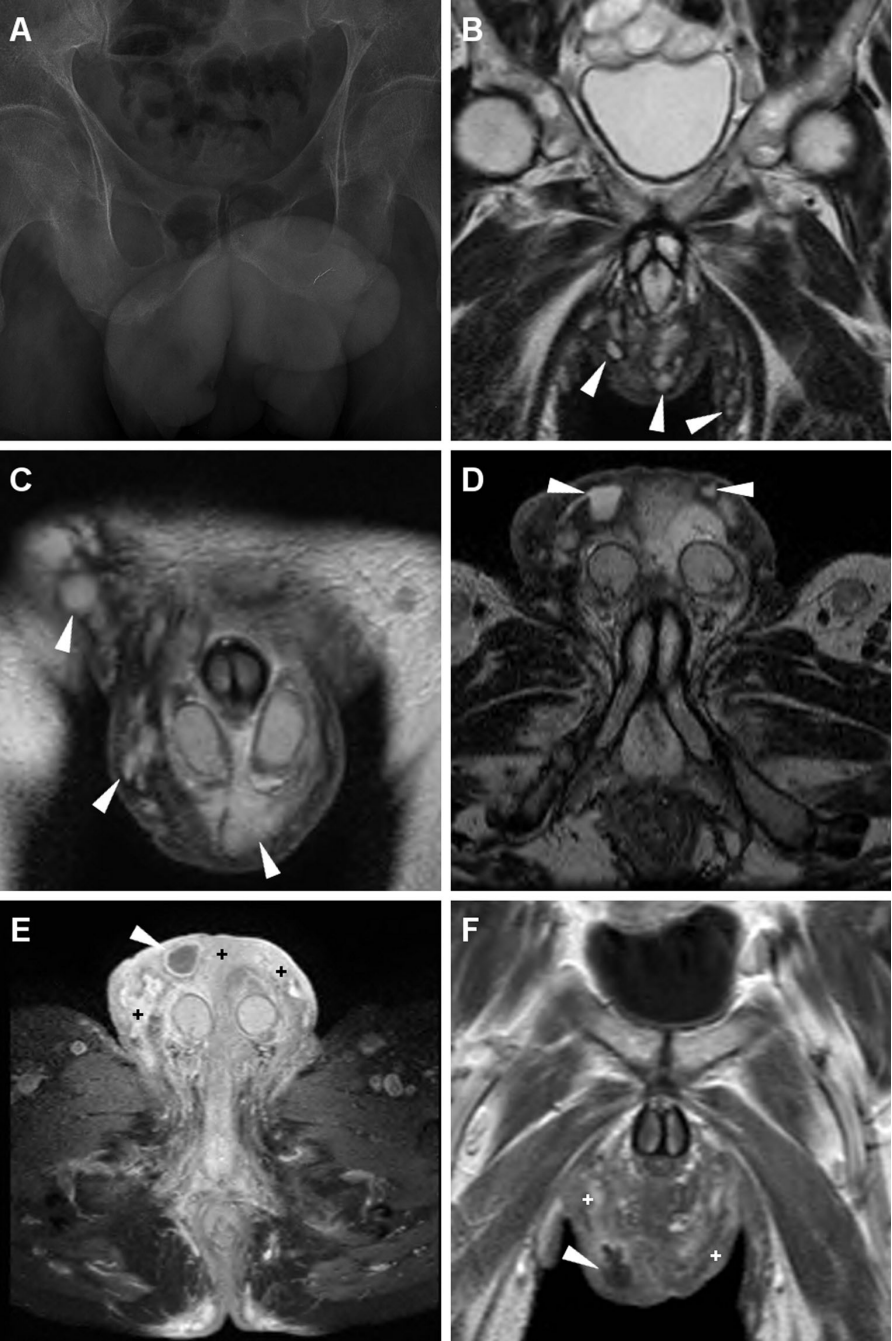
Surgically confirmed hidradenitis suppurativa in a 51-year-old man with hepatitis C, complaining of progressive, painful swelling of perineum, scrotum and penis, with thickened skin and fistulous orifices. Plain radiographs (**a**) excluded air collections in the swollen scrotum. MRI including multiplanar T2-(**b**–**d**), post- gadolinium axial fat-suppressed (**e**) and coronal (**f**) T1-weighted images depicted marked symmetrical thickening of the skin and subcutaneous planes with abnormal inflammatory signal intensity and hyperenhancement, (+ in **e**, **f**) involving the medial aspect of the thighs, perineal region and scrotum. Small purulent collections with peripheral enhancement (*arrowheads*) and inflamed inguinal lymph nodes were present. The testes (not shown) did not show appreciable abnormalities [Partially reprinted with permission from Ref. [71]]

In our experience, MRI may accurately describe the affected regions, and confirm HS over orchiepididymitis and scrotal abscess by demonstrating that tissue inflammation and abscesses are confined to the superficial planes, with a characteristic symmetric distribution and lacking communication with pelvic organs (Fig. 21) [55, 71–73].

## Conclusion

C-UTIs affecting the lower urinary tract and male genital organs are commonly encountered in patients with related risk factors such as neurogenic dysfunction or bladder outlet obstruction, obstructive uropathy from any cause, intermittent or long-term bladder catheterisation, urologic instrumentation or indwelling stent, urinary tract post-surgical modifications, chemotherapy or radiation-induced damage, renal impairment, diabetes mellitus and immunodeficiency [1].

The cross-sectional imaging features of the C-UTIs affecting the lower urinary tract and male genital organs presented herein are summarised in Table 2. Radiologists must be familiar with the CT and MRI appearance of these potentially severe disorders, which frequently require intensive in-hospital antibiotic therapy, percutaneous drainage or surgery. Furthermore, subtle imaging signs of C-UTI may be incidentally encountered in cross-sectional imaging studies performed for other indications.

**Table 2:** Cross-sectional imaging features and differential diagnoses of complicated urinary infections affecting the lower tract and male genital organs

Infectious conditions	Cross-sectional imaging signs	Key differential diagnoses
Acute infectious cystitis	Diffuse mural bladder thickening, particularly if: - marked (≥1 cm thick) - hypoenhancing - oedematous at T2-weighted MRI - increased compared to previous studies Urothelial hyperenhancement - minimally thickened - uniform, circumferential Perivesical fat inflammatory changes	Urinary bladder carcinoma Nephrogenic adenoma, malacoplakia Urinary tuberculosis Schistosomiasis w/o superimposed squamocellular carcinoma Post-chemotherapy Radiation cystitis Uncommon: cystitis cystica, cystitis glandularis, eosinophilic cystitis
Mural bladder abscess	Intramural / exophytic collection - internally hypoattenuating (10–15 HU) non-enhancing - irregular, often thick peripheral enhancement - usual site: upper bladder aspect	Infected bladder diverticulum Urinary bladder carcinoma with perivesical invasion
Emphysematous cystitis	Gas-attenuation linear changes along the bladder wall	Intraluminal air from catheterisation Enterovesical fistulisation (particularly from colonic diverticulosis or Crohn’s disease)
Prostatic abscess	Single or multifocal collection - peripheral or septal enhancement - centrally non-enhancing fluid-like Variable prostatic enlargement, urethral displacement Possible extraprostatic extension	Acute bacterial prostatitis Prostate carcinoma (particularly after treatment)
Seminal vesicle abscess	Uni- or bilateral seminal vesicle enlargement - thick irregular enhancing walls and septa - internally hypoattenuating, non-enhancing Adjacent fat inflammatory changes	Chronic infection Urinary tuberculosis Congenital cysts Metastases, rare primary tumours
Acute urethritis	Thickened penile urethra and surrounding tissues - increased T2 MRI signal intensity - corresponding increased contrast enhancement	
Periurethral abscess	Periurethral collection - internally fluid or purulent - peripheral enhancement - typical site: ventral, communicating with penile urethra Thickened, oedematous corpus spongiosum Possible further inferior extension to perineum and scrotum Possible development of necrotizing fasciitis (Fournier’s gangrene)	Urethral diverticulum
Funiculitis, epididymitis	Unilateral spermatic cord thickening - with engorged enhancing vessels Variable epididymal enlargement - increased T2 MRI signal intensity - hyperenhancing epididymis	Tuberculosis Varicocele Inguino-crural hernia Spermatocele/sperm granuloma Rare epididymal tumours, e.g. neurofibroma, metastasis
Orchitis, scrotal abscess	Unilateral testicular enlargement - decreased T1, increased T2 MRI signal intensity - increased vascularity (diffuse or “tiger skin” appearance) - loss of contrast enhancement when necrosis occurs Funiculitis and epididymitis commonly associated Abscess/pyocele: - fluid-like collections - enhancing periphery - surrounded by hypervascularised testicular parenchyma	Testicular torsion Testicular tumours, e.g. lymphoma, seminoma, germ cell tumours Necrotizing fasciitis (Fournier’s gangrene) from non-urinary source Hidradenitis suppurativa
